# Supplementary figures and images for: In silico investigation of the mechanisms underlying atrial fibrillation due to impaired Pitx2
Source: PLoS Comput Biol. 2020 Feb 25;16(2):e1007678. doi: 10.1371/journal.pcbi.1007678 (PMC7059955; doi:10.1371/journal.pcbi.1007678)

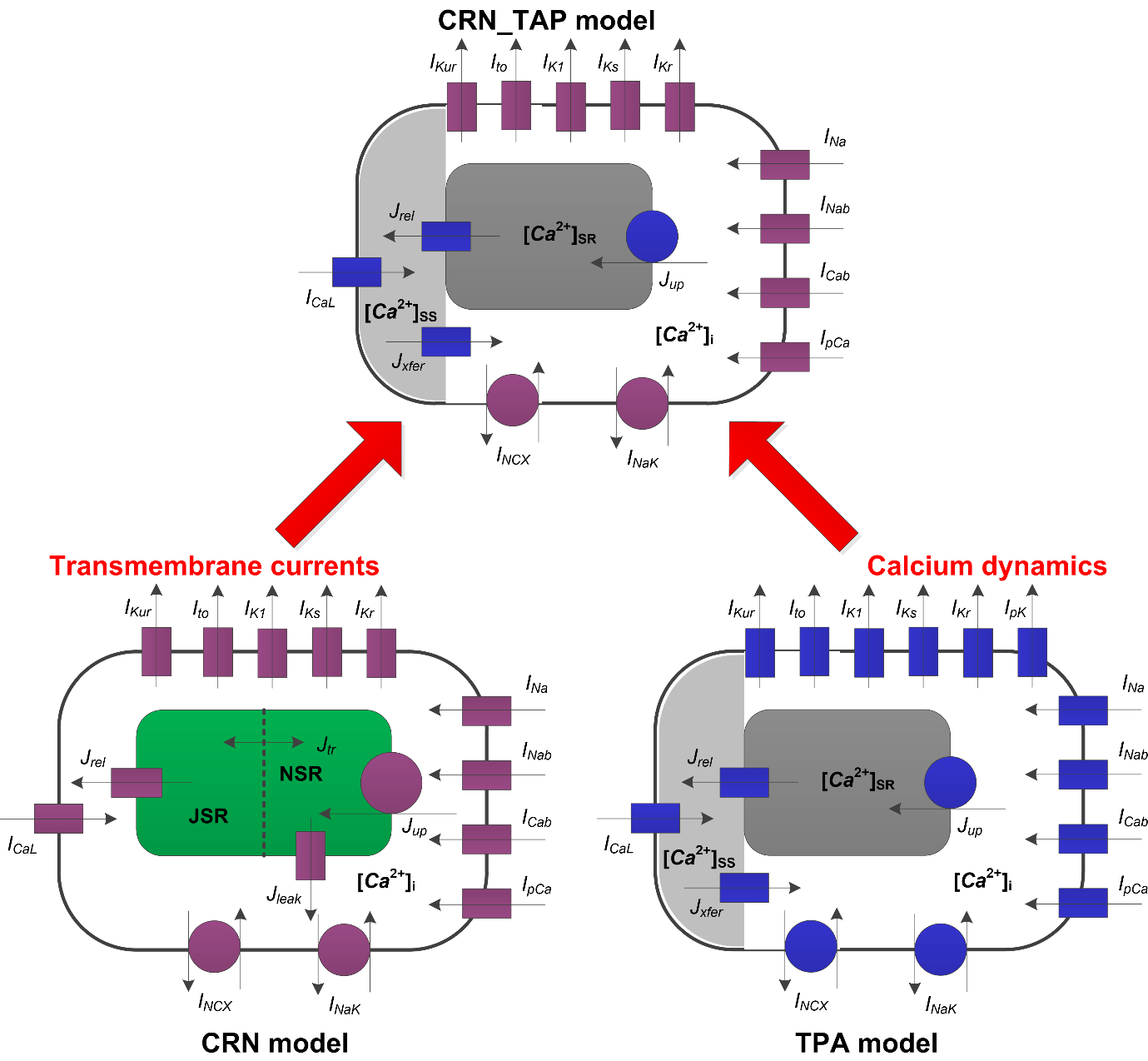

Supplement: S1 Fig — The CRN_TPA model was developed by combining the calcium handling formulations from the TPA model and the transmembrane currents of the CRN model. The cell space includes a sub-cellular compartment dyadic cleft (SS), the sarcoplasmic reticulum (SR), the cytoplasm and cell membrane. (DOCX) [file pcbi.1007678.s001.docx]

***
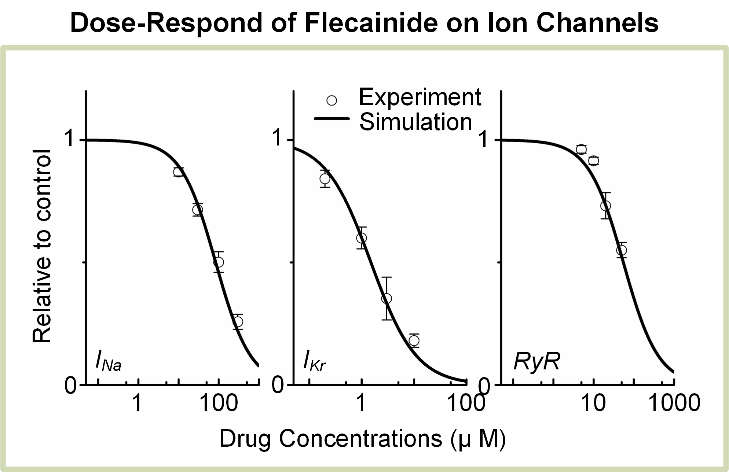
***

Supplement: S2 Fig — Dose-response for the inhibitory effects of flecainide on INa, IKr, and RyR showed that IC50 values are 84±4 μM, 1.5±0.1 μM, and 55±8 μM, respectively. Abbreviations: AAD–antiarrhythmic drug; RyR–ryanodine receptor; IC50 half-maximal inhibitory concentration. (DOCX) [file pcbi.1007678.s002.docx]

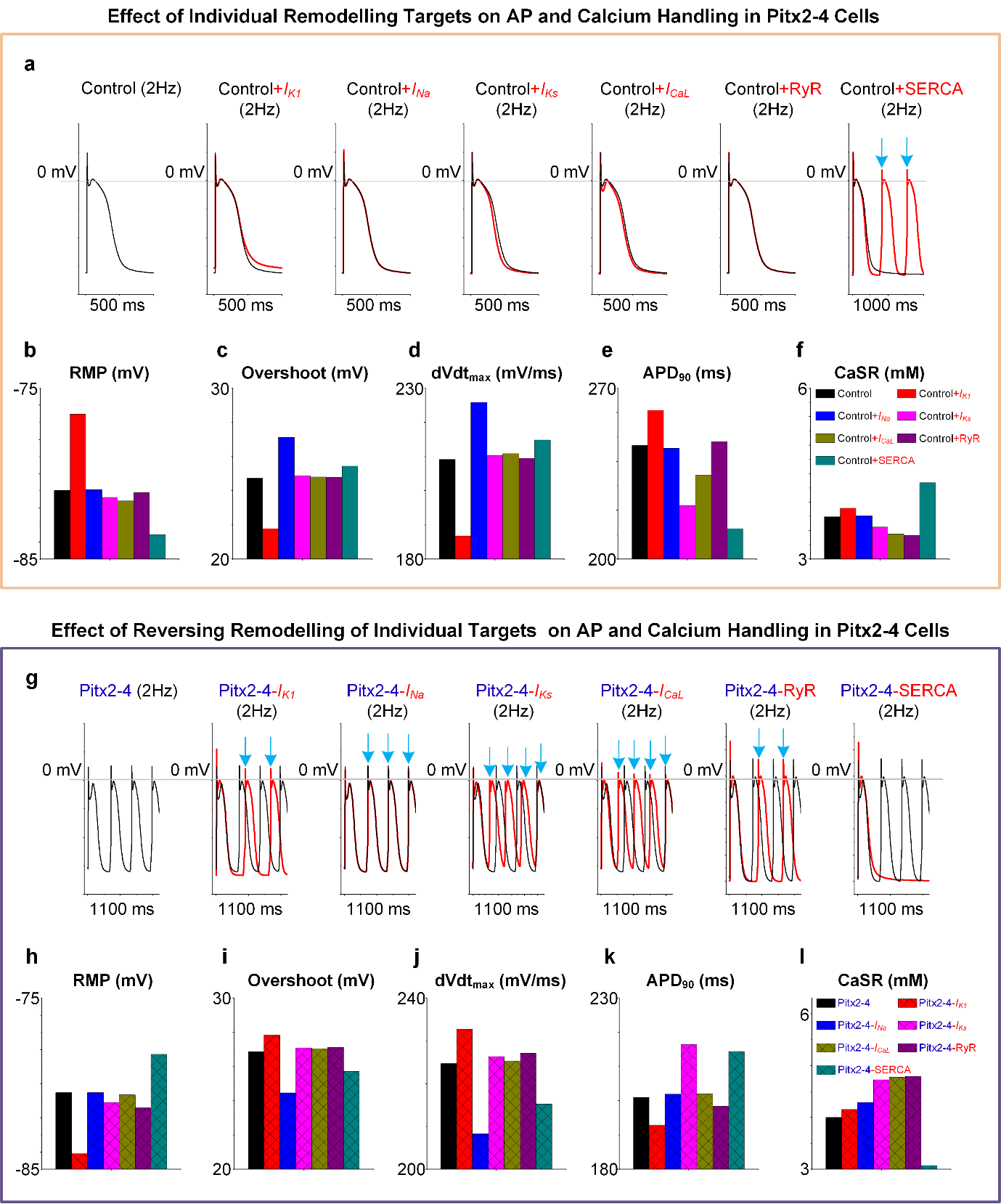

Supplement: S3 Fig — Effects of individual remodelled targets (IK1, INa, IKs, ICaL, RyR or SERCA) on AP (a), RMP (b), overshoot (c), dVdtmax (d), APD (e) and CaSR (f). Effects of reversing remodelled individual targets on key indicators (g-l). haBlue arrows indicate spontaneous delayed afterdepolarization (DAD). Abbreviations: RyR–ryanodine receptor; SERCA–calcium transport ATPase; AP–Action potential; RMP–resting membrane potential; dVdtmax–maximum upstroke velocity; APD–action potential duration; CaSR–sarcoplasmic reticulum calcium content. (DOCX) [file pcbi.1007678.s003.docx]

*
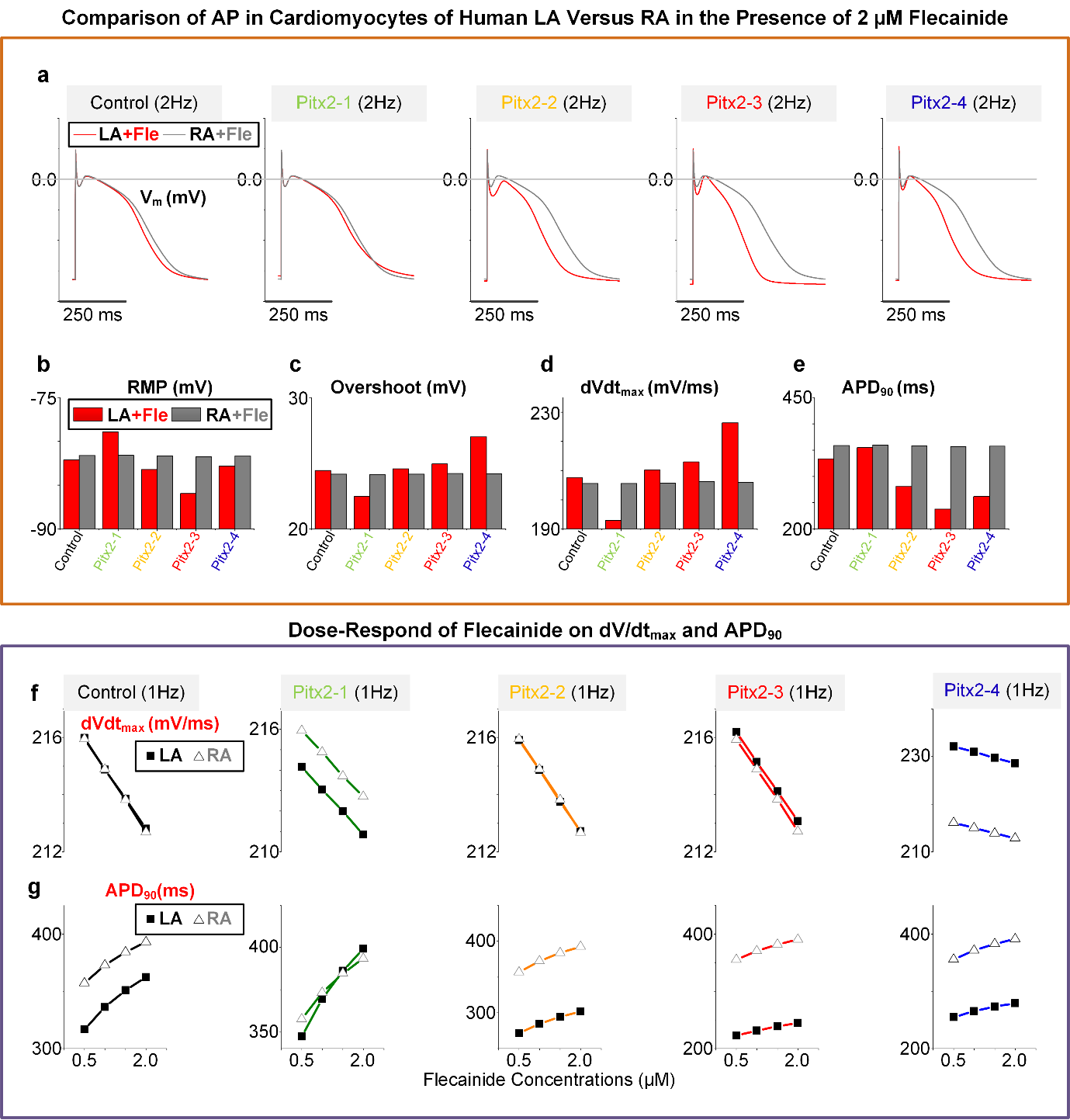
*

Supplement: S4 Fig — a, Comparison of APs of LA (red) versus RA (gray) cells in the presence of 2μM flecainide under control and four Pitx2-deficiency conditions. The main AP parameters included RMP (b), overshoot (c), dVdtmax (d) and APD (e). Within clinical dose (0.5~2 μM), flecainide reduced dVdtmax (f) and prolonged APD (g). Abbreviations: LA–left atrium; RA–right atrium; Fle–flecainide; RMP–resting membrane potential; dVdtmax–maximum upstroke velocity; APD–action potential duration. (DOCX) [file pcbi.1007678.s004.docx]

***
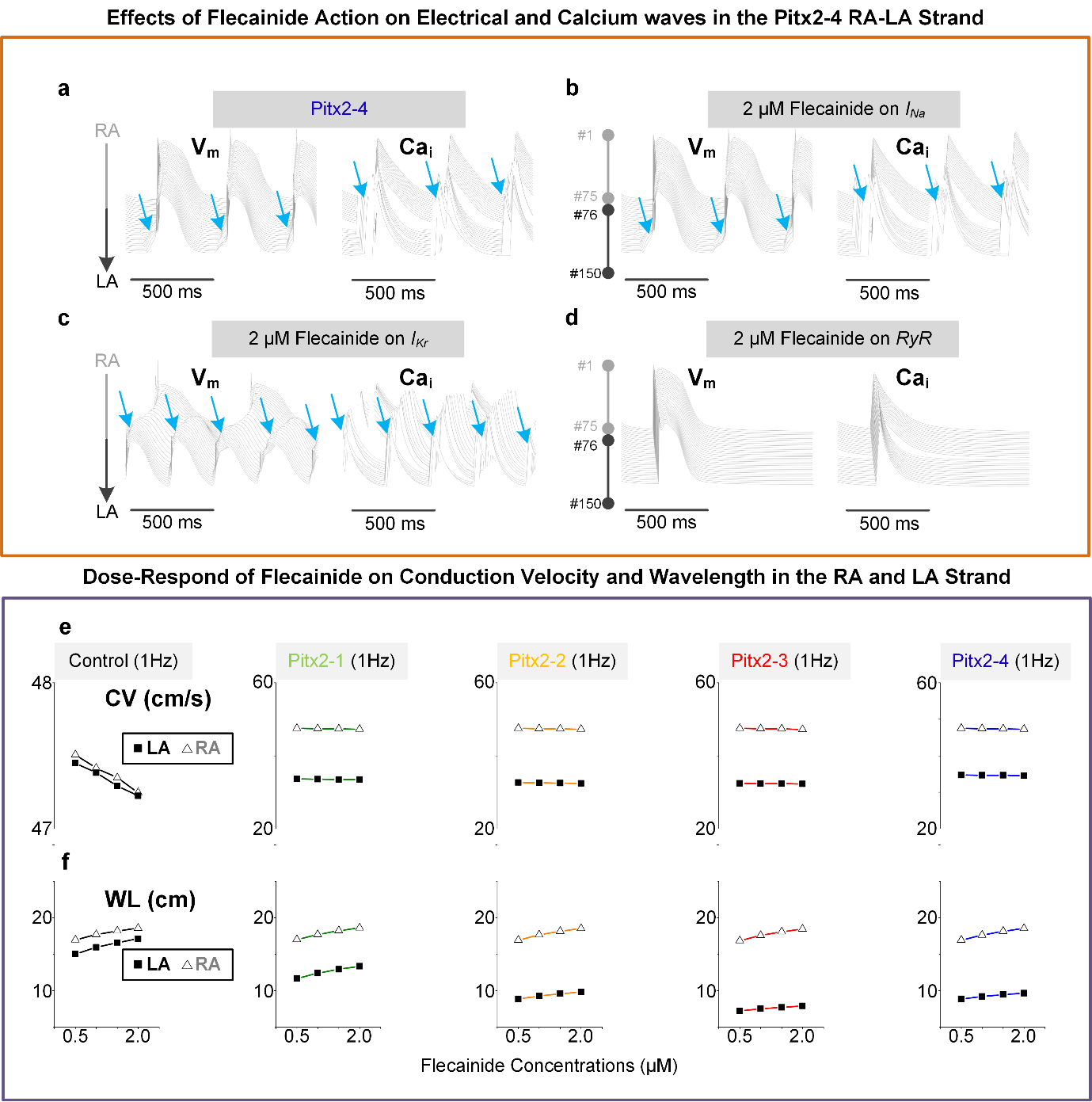
***

Supplement: S5 Fig — Compared with Vm and Cai waves in the drug-free Pitx2-4 settings (a), these waves in the presence of 2 μM flecainide on targeting INa (b), on Ikr (c) and on RyR alone (d) respectively. Blue arrows indicate spontaneous delayed afterdepolarizations, triggered action potentials and calcium transients. Within clinical dose (0.5~2 μM), flecainide reduced CV (e) and prolonged WL (f). Abbreviations: RyR–ryanodine receptor; CV–conduction velocity; WL–Wavelength. (DOCX) [file pcbi.1007678.s005.docx]

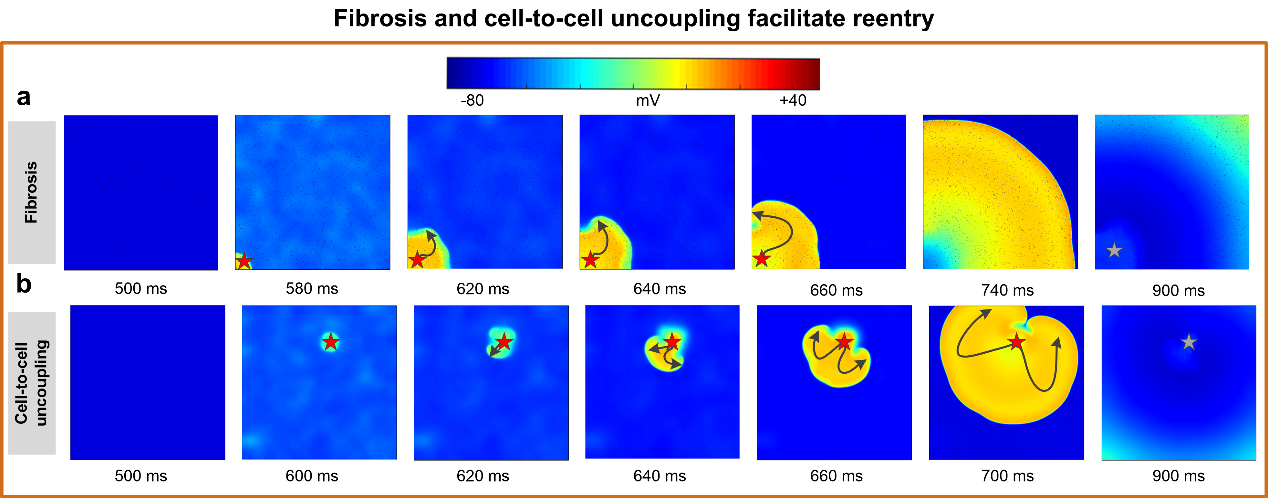

Supplement: S6 Fig — a, Simulated spontaneous ectopic activity and re-entrant waves in the tissue model with increased fibrosis. b, Simulated ectopic activity and re-entrant waves in the tissue model with cell-to-cell uncoupling. (DOCX) [file pcbi.1007678.s006.docx]

*
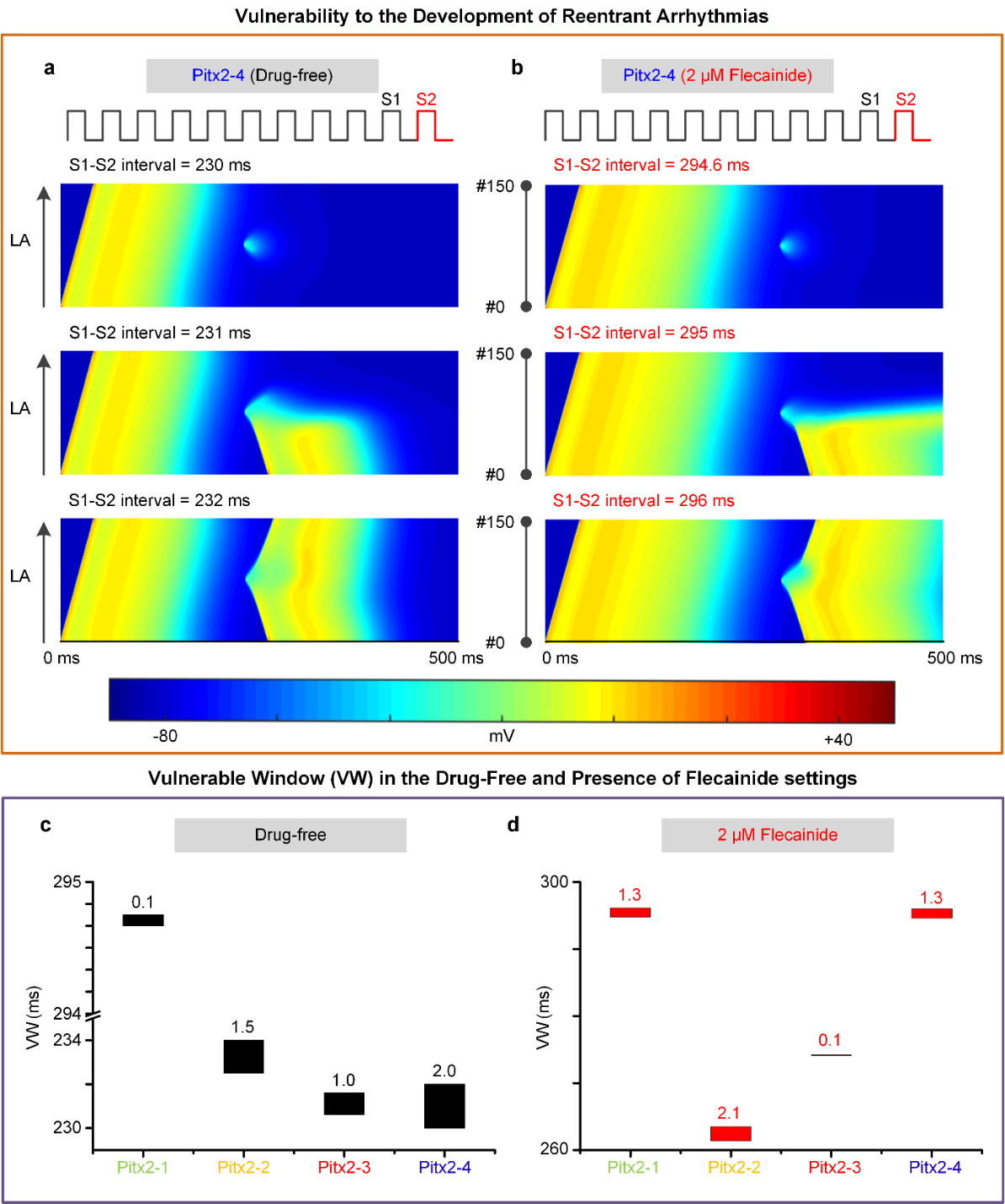
*

Supplement: S7 Fig — Bidirectional conduction block, unidirectional conduction block and bidirectional conduction in the drug-free Pitx2-4 settings (a) versus in the presence of 2 μM flecainide (b). VW under Pitx2-1, Pitx2-2, Pitx2-3 and Pitx2-4 conditions in the drug-free Pitx2-4 settings (c) versus in the presence of 2μM flecainide (d). (DOCX) [file pcbi.1007678.s007.docx]

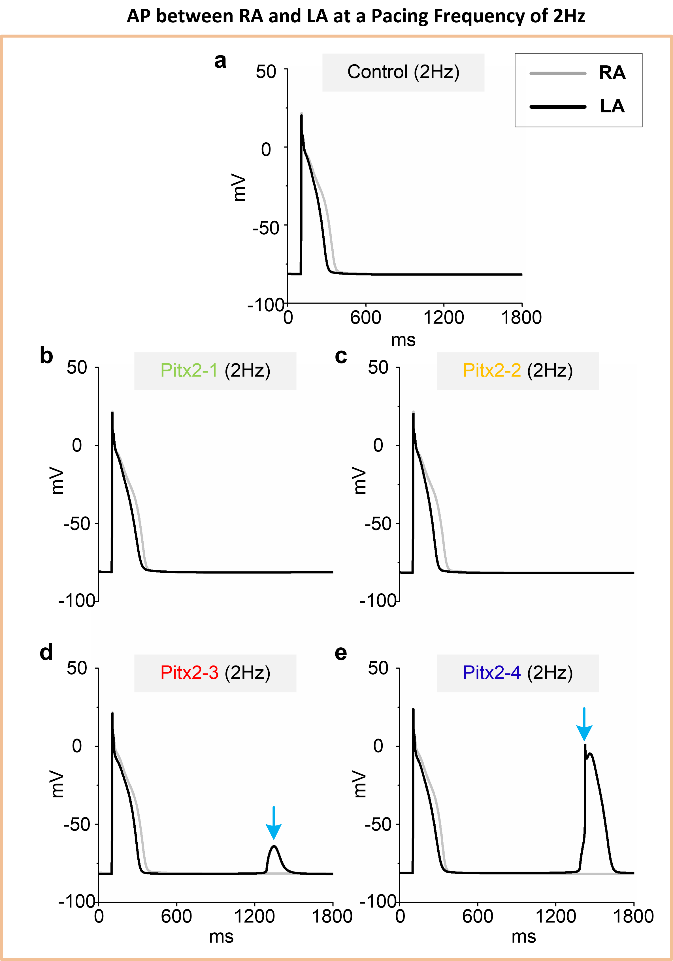

Supplement: S8 Fig — At a pacing frequency of 2Hz, AP under control, Pitx2-1, Pitx2-2, Pitx2-3 and Pitx2-4 conditions. Black and grey markers were used for LA and RA cells, respectively. Blue arrows indicate spontaneous delayed afterdepolarizations and triggered action potentials. (DOCX) [file pcbi.1007678.s008.docx]

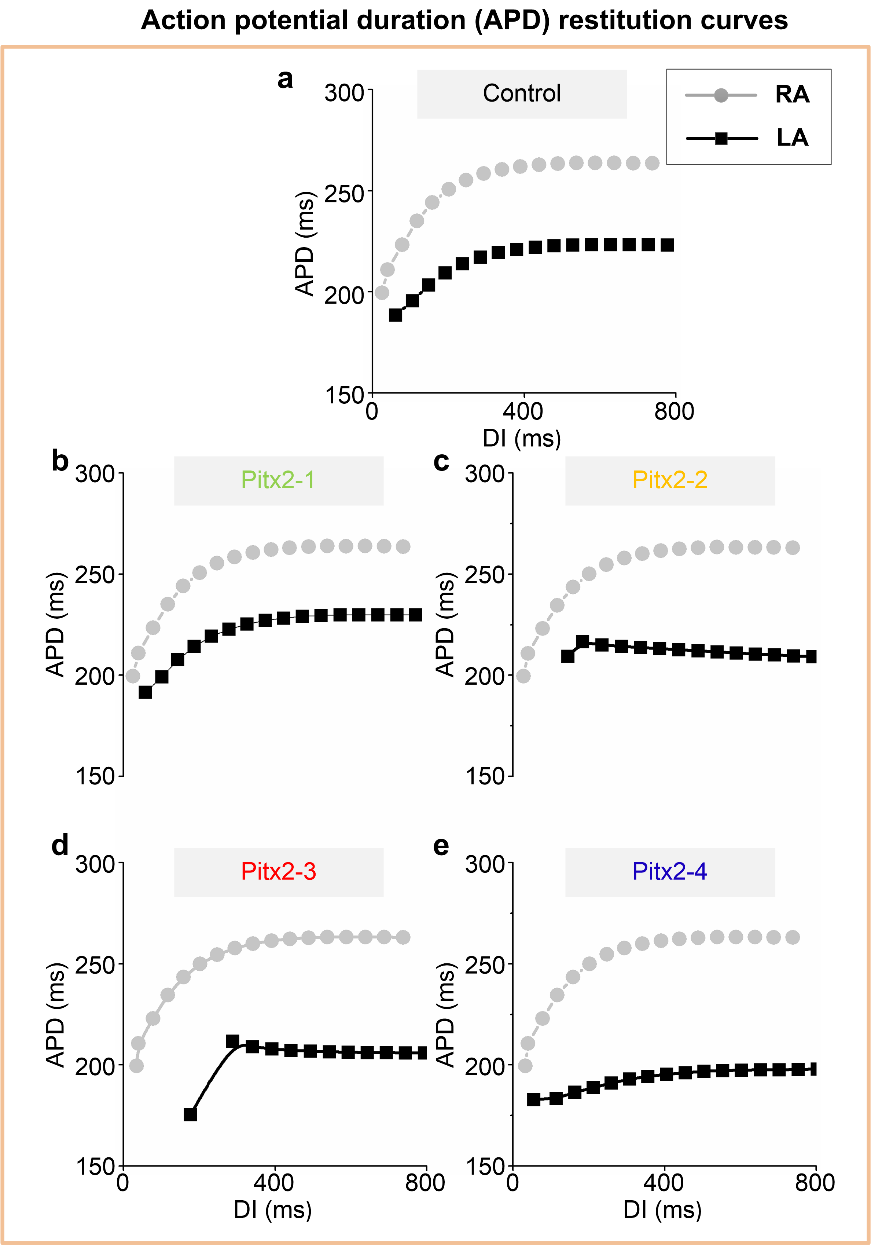

Supplement: S9 Fig — (a-e) APD restitution curves for control, Pitx2-1, Pitx2-2, Pitx2-3 and Pitx2-4 conditions. Black and grey markers were used for LA and RA cells, respectively. Abbreviations: APD–action potential duration; DI–diastolic interval; LA–left atrial cell; RA–right atrial cell. (DOCX) [file pcbi.1007678.s009.docx]

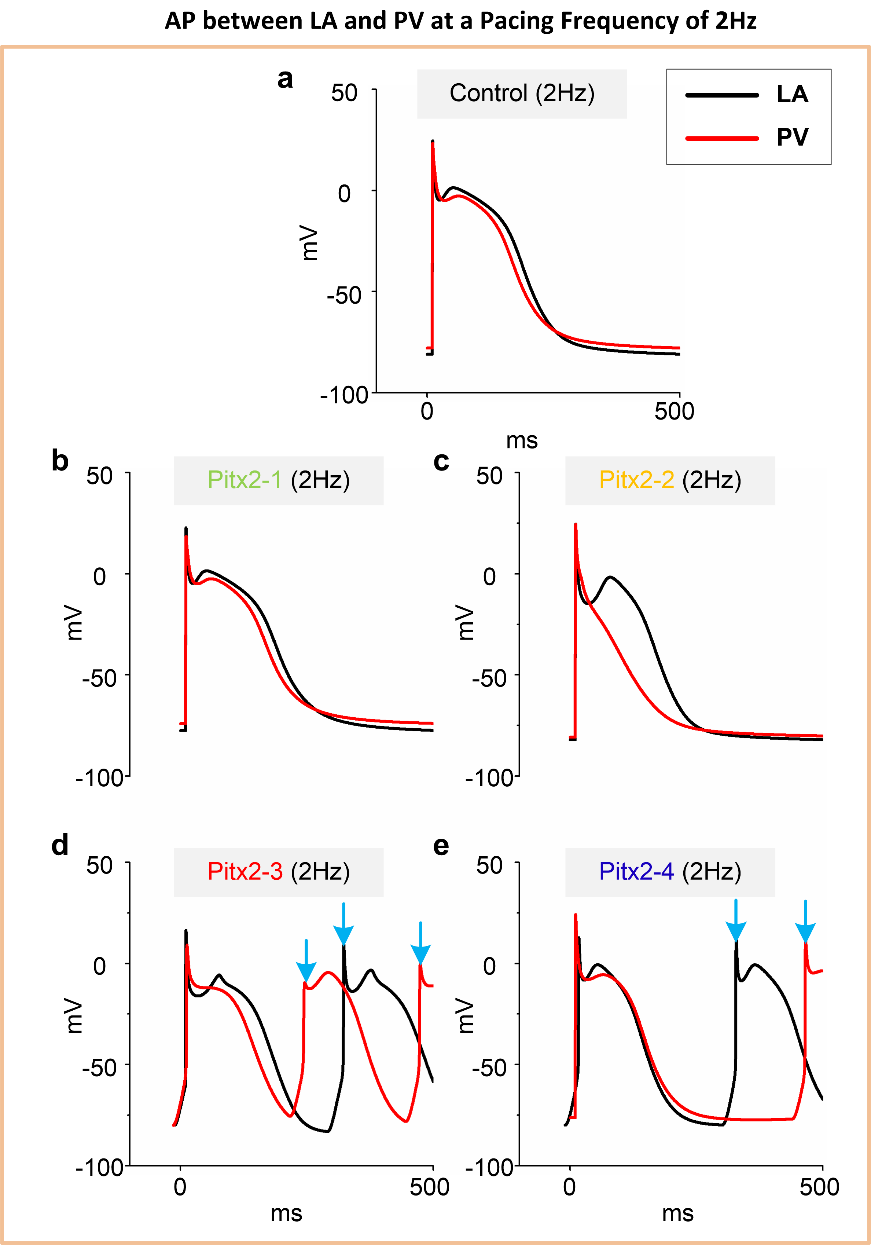

Supplement: S10 Fig — At a pacing frequency of 2Hz, AP under control, Pitx2-1, Pitx2-2, Pitx2-3 and Pitx2-4 conditions. Black and red markers were used for LA and PV cells, respectively. Blue arrows indicate spontaneous delayed afterdepolarizations and triggered action potentials. (DOCX) [file pcbi.1007678.s010.docx]
