# Supplementary material for: In silico investigation of the mechanisms underlying atrial fibrillation due to impaired Pitx2
Source: PLoS Comput Biol. 2020 Feb 25;16(2):e1007678. doi: 10.1371/journal.pcbi.1007678 (PMC7059955; doi:10.1371/journal.pcbi.1007678)
Supplement: S1 Table — (DOCX) [file pcbi.1007678.s011.docx]

|  | **ICaL(%)** | **IK1(%)** | **Ito(%)** | **IKs(%)** | **IKr(%)** | **Source** |
| --- | --- | --- | --- | --- | --- | --- |
| **RA(Control)** | 100 | 100 | 100 | 100 | 100 | - |
| **LA(Control)** | 100 | 100 | 100 | 100 | 160 | [9, 11] |
| **PV(Control)** | 75 | 70 | 50 | 180 | 100 | [10, 12] |
