# Supplementary material for: In silico investigation of the mechanisms underlying atrial fibrillation due to impaired Pitx2
Source: PLoS Comput Biol. 2020 Feb 25;16(2):e1007678. doi: 10.1371/journal.pcbi.1007678 (PMC7059955; doi:10.1371/journal.pcbi.1007678)
Supplement: S2 Table — (DOCX) [file pcbi.1007678.s012.docx]

|  | | **Control** | **Pitx2-1** | **Pitx2-2** | **Pitx2-3** | **Pitx2-4** |
| --- | --- | --- | --- | --- | --- | --- |
| **RMP** | **LA** | -80.97 | -77.45 | -82.05 | -84.38 | -83.23 |
|  | **PV** | -80.69 | -77.18 | -81.39 | -82.71 | -77.80 |
| **OS** | **LA** | 24.73 | 22.75 | 24.86 | 25.33 | 27.73 |
|  | **PV** | 24.80 | 22.62 | 24.81 | 25.27 | 24.81 |
| **TA** | **LA** | No | No | No | Yes | Yes |
|  | **PV** | No | No | No | Yes | Yes |
| **dVdt_max_** | **LA** | 209.1 | 193.8 | 211.9 | 215.8 | 231.4 |
|  | **PV** | 207.5 | 190.4 | 209.7 | 213.3 | 206.2 |
| **APD** | **LA** | 246.5 | 258.7 | 221.1 | 204.2 | 224.5 |
|  | **PV** | 238.6 | 244.3 | 183.3 | - | 207.3 |
| $\boldsymbol{\Delta APD}$ | **LA-PV** | 7.9 | 14.4 | 37.8 | - | 17.2 |

Abbreviations: LA–left atrial cells; PV-pulmonary vein cells; RMP (mV) – resting membrane potential; OS (mV) – overshoot of action potential; dVdtmax (mV/ms) –maximum upstroke velocity; APD (ms) – action potential duration; and $\Delta APD$ – APD heterogeneity. Under the Pitx2-3 condition, spontaneous delayed afterdepolarizations and triggered action potentials were induced.
