# Supplementary material for: In silico investigation of the mechanisms underlying atrial fibrillation due to impaired Pitx2
Source: PLoS Comput Biol. 2020 Feb 25;16(2):e1007678. doi: 10.1371/journal.pcbi.1007678 (PMC7059955; doi:10.1371/journal.pcbi.1007678)
Supplement: S1 Text — (DOCX) [file pcbi.1007678.s015.docx]

**Supporting text S1**

***In silico* investigation of the mechanisms underlying atrial fibrillation due to impaired Pitx2**

Jieyun Bai, Andy Lo, Patrick A. Gladding, Martin K. Stiles, Vadim V. Fedorov, Jichao Zhao

**The CRN_TPA model**

The CRN_TPA model was developed by integrating the calcium dynamics of our human atrial model (TPA)[1-3] into the Courtemanche et al. model (CRN)[4, 5] in our previous study. The intracellular structure of the CRN_TP model was compared to those of the CRN and TPA models ([**Supplementary**](https://www.nature.com/articles/srep31262#s2) **Figure S1**). No changes were made to formulations of the transmembrane currents: IKur, Ito, IK1, IKs, IKr, INa, INab, ICab, IpCa, INCX and INaK. For these formulations, we refer to their description in the original version of CRN model[5]. We have replaced the phenomenological descripation of calcium-induced calcium release in the CRN model with a reduced version of the ryanodine receptor Markov model developed by Stern et al[6]. and Shannon et al[7]. For equations and parameter values of calcium dynamics, we refer to their description in our human atrial model (TPA)[5, 8].

Restitution of the APD_90_ (APDR) was measured by using the standard dynamic method. The human atrial myocyte was firstly paced at a basic cycle length (BCL) of 1000 ms and then the BCL was progressively reduced by 5 to 50 ms. APDR curves were generated by plotting APD_90_ versus diastolic interval (DI) which was computed as BCL minus APD_90._

Regional cell models were developed based on experimentally-measured changes in maximal current density of several ionic currents[9-12] relative to the baseline right atrium (RA) model. Maximal ionic conductances were changed to generate left atrium (LA) and [pulmonary vein (PV) model](https://academic.oup.com/europace/article-abstract/16/3/416/610913)s, and can be seen in the [**Supplementary**](https://www.nature.com/articles/srep31262#s2) **Table S1**.

**Actions of flecainide on ion channels**

To investigate the anti-arrhythmic effects of the class Ic drug flecainide, we integrated the actions of flecainide on ion channels and RyR into the Pitx2-mutant computer models. Modifications to ion channels provoked by flecainide were modeled by using the standard sigmoid dose-response curve (**Supplementary Figure S4)** parametrized with *IC_50_* and Hill coefficient (*nH*=1). The values of *IC_50_* for inhibition of *I_Na_, I_Kr_* and RyR open probability were 84, 1.5 and 55 µM[13, 14], respectively.

In the present study, the SR calcium flow (*J_rel_*) of RyR is the combination of calcium-induced-calcium release flow *J_cicr_* and SR calcium leak flow *J_leak_* to reproduce triggered activity, i.e., early afterdepolarizations (EAD), delayed afterdepolarizations (DAD) and spontaneous depolarizations. Under the drug-free conditions, *J_rel_* is given by

$$\boldsymbol{J}_{\boldsymbol{rel}}\boldsymbol{=}\boldsymbol{J}_{\boldsymbol{cicr}}\boldsymbol{+}\boldsymbol{J}_{\boldsymbol{leak}} \boldsymbol{(1)}$$

$$\boldsymbol{J}_{\boldsymbol{cicr}}\boldsymbol{=}\boldsymbol{V}_{\boldsymbol{rel}}\boldsymbol{\cdot O\cdot}\left( \left[ \boldsymbol{Ca}^{\boldsymbol{2+}} \right]_{\boldsymbol{SR}}\boldsymbol{-}\left[ \boldsymbol{Ca}^{\boldsymbol{2+}} \right]_{\boldsymbol{SS}} \right) \boldsymbol{(2)}$$

$$\boldsymbol{J}_{\boldsymbol{leak}}\boldsymbol{=}\boldsymbol{V}_{\boldsymbol{sp}}\boldsymbol{\cdot R\cdot}\left( \left[ \boldsymbol{Ca}^{\boldsymbol{2+}} \right]_{\boldsymbol{SR}}\boldsymbol{-}\left[ \boldsymbol{Ca}^{\boldsymbol{2+}} \right]_{\boldsymbol{SS}} \right) \boldsymbol{(3)}$$

where *V_rel_* (0.102 mM/ms) is the maximal *J_cicr_* conductance, *V_sp_* (0.00036 mM/ms) is the maximal *J_leak_* conductance, *O* is the proportion of open RyR channels, *R* is the proportion of closed RyR channels, [*Ca*^2+^]_SR_ is the free SR calcium concentration and [*Ca*^2+^]_SS_ is the free dyadic subspace calcium concentration. In the presence of flecainide, Block=100%/$\left\{ 1+{({{[IC}_{50}]}_{i}/D)}^{nH} \right\}$ and thereby $J_{rel}=$ $Block\times J_{cicr}+(1-Block)\times J_{leak}$.

**Action potential simulations with an alternative human atrial cell model**

Comparative simulations were carried out using the human atrial cell model of Grandi *et al*. [15](GB model). The baseline GB model was modified in two ways; (i) the transmembrane currents’ formulations were replaced with those of the CRN human atrial cell model in order to reproduce EADs, DADs and triggered action potentials[16], and (ii) the maximal conductance of IK1 was increased by 200% in order to preserve the resting membrane potential under the normal condition. Action potentials were obtained at a pacing rate of 2 Hz. These Pitx2 insufficiency-induced changes in AP obtained from the modified GB model was qualitatively similar to that from the CRN_TPA model.

1. Bai J, Gladding PA, Stiles MK, Fedorov VV, Zhao J. Ionic and cellular mechanisms underlying TBX5/PITX2 insufficiency-induced atrial fibrillation: Insights from mathematical models of human atrial cells. Scientific Reports. 2018;8(1):15642. doi: 10.1038/s41598-018-33958-y.

2. Bai J, Wang K, Liu Y, Li Y, Liang C, Luo G, et al. Computational cardiac modeling reveals mechanisms of ventricular arrhythmogenesis in long QT syndrome type 8: CACNA1C R858H mutation linked to ventricular fibrillation. Frontiers in physiology. 2017;8:771.

3. Bai J, Yin R, Wang K, Zhang H. Mechanisms underlying the emergence of post-acidosis arrhythmia at the tissue level: A theoretical study. Frontiers in physiology. 2017;8:195.

4. Bai J, Lu Y, Lo AC, Zhao J, Zhang H. Proarrhythmia in the p. Met207Val PITX2c-linked familial atrial fibrillation-insights from modelling. Frontiers in Physiology. 2019;10:1314.

5. Courtemanche M, Ramirez RJ, Nattel S. Ionic mechanisms underlying human atrial action potential properties: insights from a mathematical model. American Journal of Physiology-Heart and Circulatory Physiology. 1998;275(1):H301-H21.

6. Stern MD, Song L-S, Cheng H, Sham JS, Yang HT, Boheler KR, et al. Local control models of cardiac excitation–contraction coupling: a possible role for allosteric interactions between ryanodine receptors. The Journal of general physiology. 1999;113(3):469-89.

7. Shannon TR, Ginsburg KS, Bers DM. Potentiation of fractional sarcoplasmic reticulum calcium release by total and free intra-sarcoplasmic reticulum calcium concentration. Biophysical journal. 2000;78(1):334-43.

8. Ten Tusscher KH, Panfilov AV. Alternans and spiral breakup in a human ventricular tissue model. American Journal of Physiology-Heart and Circulatory Physiology. 2006;291(3):H1088-H100.

9. Feng J, Yue L, Wang Z, Nattel S. Ionic mechanisms of regional action potential heterogeneity in the canine right atrium. Circulation research. 1998;83(5):541-51.

10. Aslanidi OV, Butters TD, Ren CX, Ryecroft G, Zhang H, editors. Electrophysiological models for the heterogeneous canine atria: computational platform for studying rapid atrial arrhythmias. 2011 Annual International Conference of the IEEE Engineering in Medicine and Biology Society; 2011: IEEE.

11. Seemann G, Höper C, Sachse FB, Dössel O, Holden AV, Zhang H. Heterogeneous three-dimensional anatomical and electrophysiological model of human atria. Philosophical Transactions of the Royal Society of London A: Mathematical, Physical and Engineering Sciences. 2006;364(1843):1465-81.

12. Colman MA, Varela M, Hancox JC, Zhang H, Aslanidi OV. Evolution and pharmacological modulation of the arrhythmogenic wave dynamics in canine pulmonary vein model. Europace. 2014;16(3):416-23.

13. Belardinelli L, Liu G, Smith-Maxwell C, Wang W-Q, El-Bizri N, Hirakawa R, et al. A novel, potent, and selective inhibitor of cardiac late sodium current suppresses experimental arrhythmias. Journal of Pharmacology and Experimental Therapeutics. 2013;344(1):23-32.

14. Watanabe H, Chopra N, Laver D, Hwang HS, Davies SS, Roach DE, et al. Flecainide prevents catecholaminergic polymorphic ventricular tachycardia in mice and humans. Nature medicine. 2009;15(4):380.

15. Grandi E, Pandit SV, Voigt N, Workman AJ, Dobrev D, Jalife J, et al. Human atrial action potential and Ca2+ model: sinus rhythm and chronic atrial fibrillation. Circulation research. 2011;109(9):1055-66.

16. Andy L, Bai J, Gladding PA, Zhao J, editors. The ionic mechanisms of triggered atrial activity under a TBX5-driven regulatory network. 2019 Computing in Cardiology Conference (CinC); 2019: IEEE.
