# Supplementary material for: In silico investigation of the mechanisms underlying atrial fibrillation due to impaired Pitx2
Source: PLoS Comput Biol. 2020 Feb 25;16(2):e1007678. doi: 10.1371/journal.pcbi.1007678 (PMC7059955; doi:10.1371/journal.pcbi.1007678)
Supplement: Attachment — Submitted filename: RebuttalLetter_15Dec_2019_Final.pdf [file pcbi.1007678.s016.pdf]

**Response**

We thank both Reviewers for taking the time to evaluate our study and provide helpful criticisms and suggestions, following which we significantly modified and improved our manuscript. To expedite the review process, we present our revisions in red-colored font and in a clean version to address the issues mentioned.

We have hence made significant modifications and improvements to our submission by providing 5 figures, 4 tables for reproducing our simulated results as described in this rebuttal letter. We have also updated 4 figures and 6 subsections in the revised manuscript.

More specifically, we have made the following major changes in the revised manuscript:

1) To address the inquire of the Reviewer #1 with regards to the effects of the RA-to-LA electrical heterogeneity on the vulnerable window ($VW_{RA-LA}$), we have measured $VW_{RA-LA}$ using an RA-to-LA tissue model with 250 RA myocytes and the other 250 LA cells. And we systematically investigated the pro-arrhythmogenic effects of Pitx2 insufficiency on electrical heterogeneity and $VW_{RA-LA}$ to further demonstrate that Pitx2 insufficiency-induced remodelling increases the $VW_{RA-LA}$ which cannot be reduced in the presence of flecainide (**new Table 2**).
2) To address the inquire of the Reviewers #1 and #2 on quantitative results, we have added a table (**new Table 2**) for summarizing multiple electrophysiology characteristics to clarify their inter-relations, and quantitative characteristics have been described in the Result part.
3) To address the concerns of the Reviewer #2 with regards to more extensive comparisons, we have investigated the pro-arrhythmogenic effects of Pitx2 insufficiency using the human atrial action potential and $Ca^{2+}$ model [Grandi et al., 2011, Circulation] to further demonstrate that Pitx2-induced SR calcium overload contributes to spontaneous delayed afterdepolarizations and triggered action potentials (**new Supplementary Figure S8**).
4) To address the concerns of the Reviewer #2 on pulmonary vein cells, we have modified our cell model to incorporate detailed the current density data specific to the left atrial (LA) and pulmonary vein (PV) cells respectively (**new Supplementary Table S1**). We also have systematically investigated the pro-arrhythmogenic effects of Pitx2 insufficiency on action potentials of PV cells. Simulated results further demonstrated that Pitx2 insufficiency-induced remodelling leads to spontaneous delayed afterdepolarizations, APD shortening and the APD heterogeneity increase (**new Supplementary Figure S10 and new Supplementary Table S2**).

We believe that these changes have significantly improved our manuscript in terms of novelty, robustness and significance. We hope that our efforts will favor a positive decision for our manuscript.

Please find our response to the specific comments made by each reviewer below.

**Reviewer #1:**

This is an interesting modelling study of the role of genetic factors in the role of atrial fibrillation (AF). Specifically, the study focuses on mechanisms underlying Pitx2 deficiency-induced AF, as well as effects of anti-arrhythmic drug (AAD) flecainide in AF patients with Pitx2 down-regulation. Pitx2 deficiency promoted AF arrhythmogenesis in single-cell and tissue atrial models through to the development of after-depolarizations and re-entrant excitations, whereas flecainide prevents both. Overall, the work is novel and interesting, but the paper could benefit from clarification and a better presentation of some of the results. Please see my specific comments and suggestions below.

**Response:** We would like to thank this reviewer for his positive endorsement and detailed suggestions and comments, which were used to improve the revised manuscript. Please see our response to the specific questions raised by this reviewer below.

1) The paper claims that "the introduction of flecainide fails to reverse the RA-to-LA electrical heterogeneity". However, it also stated that "in the presence of 2 μM flecainide, a planar wave … produced a spiral wave within a shorter VW" (compared to the flecainide-free case, by less than 1 ms). It has been shown in several studied that shorter VW is a sign of reduced electrical heterogeneity (Colman et al., 2014; 16: 416-23, Varela et al., 2016; 12: e1005245). Hence, I would expect that APD dispersion under flecainide should be reduced by 1-2 ms – was this measured? From the figures presented this is impossible to conclude, would it be possible to provide the actual numbers? It is also worth referencing the aforementioned studies, as they demonstrate the important role of increased atrial heterogeneity in AF arrhythmogenesis, and the modulating effects of AADs on the APD and APD dispersion. If flecainide in this study indeed reduces atrial heterogeneity even by a few ms, it should contribute to its anti-arrhythmic effect, as shown in the studies by Colman and Varela.

**Response:** We thank the reviewer for raising this very important question and agree with this reviewer that VW or APD dispersion needs more explicitly explored in the revised manuscript.

In the original manuscript, the vulnerable window (VW) wasn't measured in the RA-to-LA tissue model, but it was measured in a 1D LA tissue model and thereby it was not an index of the RA-to-LA electrical heterogeneity. We apologize for the confusion.

Taking into account what Reviewer #1 has suggested, we have measured $VW_{RA\text{-}LA}$ using the RA-to-LA tissue model. Furthermore, we provided the values of APD, APD dispersion (ΔAPD) and $VW_{RA\text{-}LA}$ to explain that whether flecainide can reduce atrial heterogeneity. As listed in the new **Table 2** in the revised Page 26, Pitx2 insufficiency-induced remodelling increased the RA-to-LA electrical heterogeneity (indexed by ΔAPD and $VW_{RA\text{-}LA}$) which was not reduced in the presence of 2 μM flecainide.

*Note: This is a part of Table 2 for summarizing the RA-to-LA electrical heterogeneity.*

| | *$APD_{RA}$* | *$APD_{LA}$* | *ΔAPD* | *$VW_{RA\text{-}LA}$* |
|---|---|---|---|---|
| *Control* | *292.5* | *246.5* | *46.0* | *0.7* (293.8-294.5) |
| *Pitx2-4* | *291.5* | *224.5* | *67.0* | *3.0* (291.8-294.8) |
| *Pitx2-4+Fle* | *357.5* | *261.6* | *95.9* | *17.0* (332.8-349.8) |

Here, APD: action potential duration; VW: vulnerable window; RA/LA: right/left atrium; Fle: Flecainide.

In the revised MS, we have added discussion with regard to the modulating effects of AADs on the RA-to-LA electrical heterogeneity and cited the two interesting papers in the revised manuscript as suggested by the reviewer (Page 16, Lines 20-25, and Page 16, Lines 1-3).

**“***However, flecainide also had adverse effects in Pitx2-induced AF. On the one hand, both of Pitx2-induced structural remodeling and the action of flecainide on INa can slow CV and facilitate re-entry. On the other hand, both of Pitx2-induced electrical remodelling and the actions of flecainide on ion channels may increase the RA-to-LA electrical heterogeneity, promoting APD dispersion and thereby unidirectional conduction block indexed by VWRA-LA [29, 30]. These findings support the notion that flecainide is not recommended in patients with structural heart disease due to high proarrhythmic risk based on the clinical findings[41]. Thus, flecainide may be an effective antiarrhythmic drug for the treatment of Pitx2-induced AF patients without the structural disease[40, 42, 43].”*

2) Four genetic variants Pitx2-1, -2, -3, -4 are considered in single-cell, 1D and 2D tissue models and multiple electrophysiology characteristics are measured in each case. Due do the large number of cases considered, it’s not easy to see the general picture. The paper could benefit from one or more tables summarizing these multiple characteristics and their inter-relations (e.g., APD and VW increased/decreased, triggered activity or re-entry present/absent, etc).

**Response:** We would like to thank this reviewer for his or her these beneficial suggestions. The new **Table 2** in the revised Page 26 for summarizing multiple electrophysiology characteristics has been added in the revised MS. In details, these characteristics includes RMP, OS, dVdtmax, TA (present/absent), APD, ΔAPD, CV, ΔCV, WL, ΔWL, TA in the 1D model (present/absent), $VW_{RA-LA}$, 2D TA (present/absent) and 2D Reentry (present/absent)**.** For simplification, we provided a part of **Table 2** for summarizing inter-relations of electrophysiology characteristics.

*Note: This is a part of Table 2 for summarizing inter-relations of electrophysiology characteristics.*

| | | Control | Pitx2-1 | Pitx2-2 | Pitx2-3 | Pitx2-4 | *Pitx2-1+Fle | *Pitx2-2+Fle | *Pitx2-3+Fle | *Pitx2-4+Fle |
|---|---|---|---|---|---|---|---|---|---|---|
| 0D | TA(LA) | No | No | No | Yes | Yes | No | No | No | No |
| | ΔAPD | - | ↓ | ↑ | ↑ | ↑ | ↓ | ↑ | ↑ | ↑ |
| 1D | ΔCV | - | ↑ | ↑ | ↑ | ↑ | ↓ | ↓ | ↓ | ↓ |
| | ΔWL | - | ↑ | ↑ | ↑ | ↑ | ↑ | ↑ | ↑ | ↑ |
| | TA | No | No | No | Yes | Yes | No | No | No | No |
| | $VW_{RA-LA}$ | - | ↑ | ↑ | ↑ | ↑ | ↑ | ↑ | ↑ | ↑ |
| 2D | Reentry | - | - | - | - | Yes | - | - | - | No |
| | TA | - | - | - | - | Yes | - | - | - | No |

Here, TA: triggered activity; APD: action potential duration; WL: wavelength; VW: vulnerable window; LA: left atrium; Fle: Flecainide. * values compared against corresponding Pitx2 cases.

3) Atrial heterogeneity itself is modelled quite simplistically, which is a major limitation. First, there is a sharp transition between the RA and LA, which is non-physiological, since there is no direct conduction between the chambers (and even if there was, it would not have been a sharp charge). Moreover, RA-

LA differences are accounted for by changing only one ionic current, IKr. However, ionic difference between the RA and LA are more diverse (Colman et al., J Physiol. 2013; 591: 4249-72). This should at least be mentioned in Limitations.

**Response:** We would like to thank this reviewer for his suggestions. The limitation of modelling atrial heterogeneity in this study has been added in the revised MS (Page 18, Lines 5-13):

*"Furthermore, we modelled atrial heterogeneity in the RA-LA tissue by considering the difference in IKr[26]. However, the ionic differences between the RA and LA are more diverse [57, 58], and this intrinsic heterogeneity should be considered. In addition, our idealized RA-LA tissue model did not include transition regions between RA and LA, electrical properties of transition regions, realistic geometry of these subregions, and fiber orientation. These limitations lead to a sharp transition between the RA and LA in our idealized tissue model. Special attention should be paid to explain our simulated results, and the effects of these factors on perpetuation and maintenance of re-entrant excitation should be further investigated[50]."*

4) Whereas all four Pitx2 models are characterised by shorter APD in the LA compared to RA, other AP characteristics change differently between the chambers and Pitx2 models. For example, RMP is higher in the LA in Pitx2-1 and Pitx2-4, but higher in the RA in Pitx2-2 and Pitx2-3; moreover, these difference are rate-dependent (Figure 2). It's worth discussing what are ionic mechanisms of such differences, and which Pitx2 model may be most physiological.

**Response:** We thank this reviewer for raising these very important questions. As suggested, we have discussed these three issues: 1) the role of electrical heterogeneity (including APD) between RA and LA in arrhythmogenesis (Page 15, Lines 5-25); 2) ionic mechanisms underlying Pitx2 insufficiency-induced AP phenotype (including ectopic depolarizations, abbreviated APs, elevated RMP and membrane excitability) in different Pitx2 models (Page 14, Lines 10-25 and Page 15, Lines 1-3), and 3) which Pitx2 model may be most physiological (Page 14, Lines 5-10).

**The Discussion part (Page 15, Lines 5-25):**

*"Increased electrical heterogeneity between RA and LA due to down-regulated Pitx2 expression implicated in the initiation and maintenance of reentrant arrhythmias.[36] Since the ratio of Pitx2 between LA and RA is 100:1 in the human atria,[8] we assumed that the extent of remodeling after Pitx2 deletion is dependent on the amount of Pitx2. In this study, increased electrical heterogeneity may result from the difference in Pitx2-induced remodeling between LA and RA. In our study, we observed that electrical remodeling induced by Pitx2 deficiency causes APD abbreviation and ectopic depolarizations in LA myocytes, but not in RA myocytes. Thus, Pitx2 insufficiency can cause an elevated difference in electrical properties between RA and LA cells, increasing repolarization dispersion in tissue and thereby susceptibility to the development of reentry[29, 37, 38]. Furthermore, Pitx2 deficiency can also cause LA structural remodeling by regulating cardiac structural genes, increasing electrical and structural heterogeneity between the two atrial chambers. Finally, our computer simulation studies suggest that the introduction of flecainide can suppress triggered activity but fail to reverse the RA-to-LA electrical heterogeneity. The presence of increased fibrosis and decreased cell-to-cell coupling under structural*

*remodeling further facilitates slow conduction, WL abbreviation, triggered activity and the initiation of reentrant drivers, increasing susceptibility to AF. It is known that Pitx2-dependent network regulates cardiac structural genes (Gja1, Gja5 and Dsp)[18] and Pitx2-induced structural remodeling leads to fibrosis and cell-cell uncoupling[39]. Also, structural remodeling due to the left-sided Pitx2 expression increases the intrinsic heterogeneity (i.e., CV and WL) between RA and LA, facilitating the development of reentry (**Figure 6a**).”*

**The Discussion part (Page 14, Lines 10-25 and Page 15, Lines 1-3):**

*“Ionic mechanisms underlying Pitx2 insufficiency-induced AP phenotype in different Pitx2 models were investigated by incorporating each ionic remodelling into the control model and reversing each remodeled target separately in the Pitx2-4 model. Consistent with experimental data on Pitx2 insufficiency-induced electrical remodelling[14, 16-18], remodeled targets considered in the Pitx2-4 model include $I_{K1}$, $I_{Na}$, $I_{CaL}$, $I_{Ks}$, RyR and SERCA. In these remodelled targets, we observed that downregulated $I_{K1}$ resulted in a more positive RMP and a prolonged APD, upregulated $I_{Na}$ contributed to a high overshoot and $dV/dt_{max}$, the $I_{Ks}$ increase and (or) the $I_{CaL}$ decrease led to a reduction in APD, and increased SR calcium load due to enhanced SERCA function caused triggered APs. On the one hand, our simulated results support the notion that down-regulation of $I_{CaL}$ and up-regulation of $I_{Ks}$ are hallmarks of electrical remodelling in AF patients and mainly cause APD shortening[32, 33]. On the other hand, our results demonstrate that enhanced SERCA function can increase the incidence of spontaneous SR calcium release events, in line with the experimental observation in AF patients[34, 35]. The calcium extrusion due to the spontaneous SR calcium release via $I_{NCX}$ can contribute to phase-4 depolarizations. In addition to the inward $I_{NCX}$ due to enhanced SERCA function, the remodelled $I_{K1}$ and $I_{Na}$ modulate RMP and membrane excitability[21], but our data and other studies[34, 35] suggest that triggered APs are mainly due to SR calcium load resulted from enhanced SERCA function.”*

**The Discussion part (Page 14, Lines 5-10):**

*“Electrical remodelling due to Pitx2 insufficiency accounts for ectopic depolarizations[18, 21] and abbreviated APs[12], contributing to AF, but the ionic mechanisms underlying these changes in AP characteristics remain unclear. In the present study, we incorporated variations in experimental data into four computer models (Pitx2-1, Pitx2-2, Pitx2-3 and Pitx2-4). In these models, the Pitx2-4 model took into account all identified regulators and best represented Pitx2 deficiency-induced electrical remodeling.”*

5) I think some phrasing throughout the paper can be improved. First, the title “Resolving the mechanism…” makes an excessively strong statement and should be toned downed. Similarly, a statement about “demystifying” the role of Pitx2 in Introduction should be moderated. Then the second para of Introduction uses a lot of terms and jargon specific to a narrow research field and is difficult to follow – please re-write using more general terms. Finally, in what sense are rs2200733 and rs10033464 “the most important variants”?

**Response:** We would like to thank this reviewer for his detailed suggestions and comments. We have modified accordingly based the reviewer’s suggestions and it includes 1) the title “*Resolving the*

*mechanism underlying increased susceptibility to atrial fibrillation in patients with impaired Pitx2*" was changed to "*In silico investigation of the mechanisms underlying atrial fibrillation due to impaired Pitx2*"; 2) *"demystify the functional role of Pitx2-induced remodeling on elevated atrial arrhythmogenesis" was modified to "investigate the functional role of Pitx2-induced remodeling in atrial arrhythmogenesis";* and 3) our introduction part using more general terms has been revised (Page 4, Lines 7-25 and Page 5, Lines 1-4). Finally, we apologize for this mistake (The most important variants (rs2200733 and rs10033464)) and have corrected all mistakes throughout the revised manuscript.

**The Introduction part (Page 4, Lines 7-25 and Page 4, Lines 1-4):**

*"These single nucleotide polymorphisms rs2200733 and rs10033464 were firstly identified in European, Chinese and Japanese populations on chromosome 4q25[5]. The gene-poor 4q25 region harbors the Pitx2 homeobox gene, which has been implicated in AF predisposition[2, 3, 6].*

*Pitx2 plays an important role in a left-sided signaling pathway that establishes the left-right asymmetry of the heart[7, 8].Pitx2 encodes 3 isoforms (Pitx2a, Pitx2b and Pitx2c) and the Pitx2c isoform promotes the embryonic development of cardiac left–right asymmetry, with levels in right and left atriums being 1:100[9]. The atrial-selective transcription factor Pitx2 is an upstream transcriptional regulator of atrial electric function and cardiogenesis[10, 11]. Complete loss of Pitx2 function can result in malformation of the pulmonary veins that are well-known sites for ectopic activity promoting spontaneous AF[8]. Pitx2 loss-of-function mouse mutants displayed abnormal electrocardiograms with atrioventricular block, irregular R-R intervals and low voltage P waves[12, 13]. In the Pitx2-mutant atrial myocytes, a significantly more depolarized resting membrane potential (RMP)[13, 14], action potential duration (APD) shortening[9, 15], and abnormalities in calcium handling[16, 17] were observed. Furthermore, expression array analyses identified genes related to calcium handling, gap junctions and ion channels affected by the reduced expression of Pitx2, mediating AF risk in carriers of common gene variants[9, 12, 13, 15, 17-19]. However, the precise AF pathophysiology under reduced Pitx2 remains unclear. Population-based studies have assessed the influence of common SNPs related to AF on the response to antiarrhythmic drug (AAD) therapies and showed that carriers of the variant allele at rs10033646 on chromosome 4q25 (Pitx2) responded favorably to the class I AAD (flecainide)[20-24]. The possible reasons behind this remain elusive."*

6) Figure 5d and f are difficult to interpret (f in particular). Are we looking at the voltage? Do all cells in panels f fire at once (going from blue to yellow) and then become desynchronised (mixture of blue and yellow in the last two panels)? These patterns look non-physiological. Why in panels d the patterns are different, with desynchrony seen already in the second panel? Please also see comment 2 above about better systemising all results in the paper.

**Response:** We apologize for the confusion. This reviewer is right that Figure 5d and f show the voltage. For Figure 5, a 500×500 square tissue model with randomly distributed normal myocytes, Pitx2-4 cells and fibrosis was designed to simulate the synchronization of triggered beats and investigate the conditions of arrhythmia induction. AP synchronization in the tissue model was simulated by a stimulus applied to the entire tissue at the beginning (t=0 ms) according to de Lang's method. All cells in panels (c-f) fire once

(going from blue to yellow and blue at t =500 ms) and then desynchronized due to mixture of normal (no triggered APs) and Pitx2-4 (exhibiting triggered APs) cells. Panels in the Figure 5d show snapshots of initiation and conduction of spontaneous ectopic activity taken at 500ms (first panel), 590ms (second panel), 930ms (third panel) and 1000ms (fourth panel). After the stimulus applied to the entire tissue (t=0 ms), all the cells (including normal and Pitx2-4 cells), all cells recovered from an action potential at t=500 ms, multiple subregions (due to Pitx2-4 exhibiting triggered APs) generated triggered activities at t=590 ms, ectropic beats occurred at only two subregions at t=930 ms and propagated to the other regions at t=1000 ms. Therefore, the patterns are different. To clarify this issue, we have added further information in the Methods part (Page 8, Lines 5-14) and provide explanations (Lines 7-15, Page 24) in the Figure 5 legend to improve readability.

**The Methods part (Page 8, Lines 5-14):**

*"To illuminate the spatiotemporal dynamics of triggered activity, a 500×500 square tissue model with randomly distributed normal myocytes, Pitx2-4 cells and fibrosis was designed to simulate synchronization of triggered beats and investigate the conditions of arrhythmia induction. Five different scenarios (#1, #2, #3, #4 and #5) were considered here for investigating effects of Pitx2-4 cells, fibrosis and cell-to-cell uncoupling on the inducibility of ectopic activity. D was set to be 100% for the #1, #2 and #3 conditions, and 30% for the #4 and #5 conditions, respectively. The ratios of normal cells, Pitx2-4 cells and fibrosis were set to be 80:20:0, 40:60:0, 78:20:2, 80:20:0 and 78:20:2, for the #1, #2, #3, #4 and #5 conditions, respectively. AP synchronization in the tissue model was simulated by a stimulus applied to the entire tissue at the beginning according to de Lang's method[31]."*

**Figure 5 legend (Page 23, Lines 2-15):**

*"Note: For the #1 scenario, the diffusion coefficient (D) was set to be 100% and the ratio of normal cells, Pitx2-4 cells and fibrosis was set to be 80:20:0. For the #2 scenario, the number of Pitx2-4 cells was increased to 40% and thereby the ratio of different cell types was 40:60:0. And D was set to be 100%. For the #3 scenario, D was reduced to 30% to model cell-to-cell uncoupling and the ratio of different cell types was set to be 80:20:0. For the #4 scenario, fibrosis was increased to 2% and thereby the ratio of different cell types was 78:20:2. And D was set to be 100%. For the #5 scenario, fibrosis was increased to 2% and D was reduced to 30%. And the ratio of different cell types was 78:20:2."*

**Reviewer #2:**

This study by Bai et al. uses computational simulations to investigate the effects of impaired Pitx2 properties on atrial fibrillation susceptibility and reentry properties. The study uses single cell, 1D and 2D simulations to investigate these effects, comparing four different Pitx2 cell model representations. The effects of flecainide are then simulated on one of the cell model variants. This represents a nice and well-designed use of simulation to investigate AF mechanisms, incorporating available experimental data. I have a few main comments, and some minor comments for improving the presentation of the study.

**Response:** We would like to thank this reviewer for his or her very positive comments. In the revised manuscript, we have provided additional results as requested by the reviewer. Please see our detailed responses to specific questions raised by this reviewer below.

**Main comments:**

1) The results of this study may be highly dependent on the choice of atrial cell model. The authors show that calcium dynamics are important in predicting the effects of pitx2. Please could the authors justify their choice of cell model, and if possible predict how their findings would change with the use of a different atrial cell model?

**Response:** We thank the reviewer for raising this very important question and agree that it is important to show our results are independent on the choice of atrial cell models. Firstly, the CRN-TPA model used in the present study was chosen as it is able to reproduce human AP morphology, triggered activity, APD rate dependence and excitation dynamics for studying reentrant arrhythmias in human atrial tissue. This model was developed base on the CRN model and was validated in our previous study [Bai et al., Scientific reports, 2018]. Secondly, an alternative human atrial cell model, the Grandi et al cell model which can reproduce triggered activities, has also been used to investigate the effects of Pitx2-induced remodelling on action potential for comparison. Similar results (spontaneous delayed afterdepolarizations and triggered action potentials under the Pitx2-3 and Pitx2-4 conditions) were observed and we have provided the new **Supplementary Figure S8** and corresponding text at Page 7 of the revised Supplementary Materials.

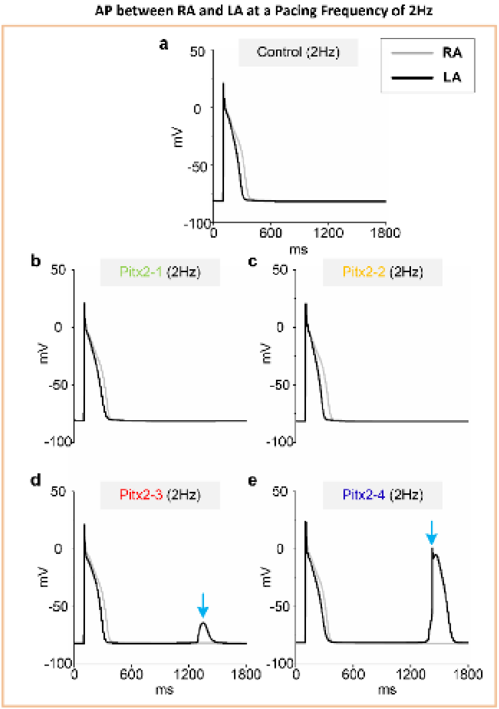


***Supplementary Figure S8. Simulated action potentials (AP) of left atrial (LA) and right atrial (RA) cells under controls and Pitx2-induced remodelling conditions using the Grandi et al. model,.*** *At a pacing frequency of 2Hz, AP under control, Pitx2-1, Pitx2-2, Pitx2-3 and Pitx2-4 conditions. Black and gray markers were used for LA and RA cells, respectively. Blue arrows indicate spontaneous delayed afterdepolarizations and triggered action potentials.*

2) How do you think pulmonary vein cell response would compare to the left atrial cell model findings here? Could you perform a subset of simulations for a cell model with ionic properties modified to match pulmonary vein cells and see how DAD incidence and action potential properties change?

**Response:** We would like to thank this reviewer for his or her detailed comments. Here, we investigated pulmonary vein cell response according to the suggestions of this reviewer.

According to the work of Colman et al. (Colman et al., 2014; 16: 416-23), our cell model was modified to incorporate detailed the current density data specific to the left atrial (LA) and pulmonary vein (PV) cells. The resultant APs in the PV cell has a shorter APD and more positive resting potential than the LA cell (Supplementary Figure S10(a) and Supplementary Table S2). Under the Pitx2-3 and Pitx2-4 conditions, spontaneous delayed afterdepolarizations and triggered action potentials were observed in PV and LA cells (Supplementary Figure S10(d-e)). Moreover, APD heterogeneity ($\Delta APD$) was increased under Pitx2-induced remodelling conditions (Supplementary Table S2).

In the present study, we focused on the proarrhythmic effects of impaired Pitx2 on RA and LA cells, and the pulmonary vein cell response was beyond the scope of this study. Therefore, these results were not included in the revised MS.

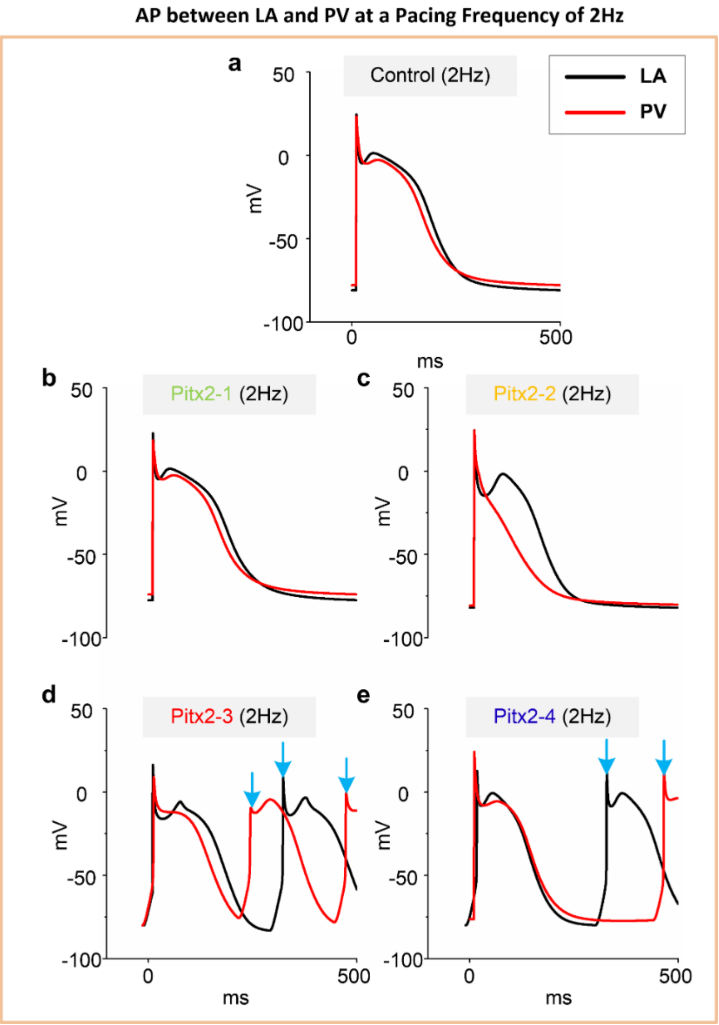


***Supplementary Figure S10. Simulated action potentials (AP) of left atrial (LA) and pulmonary vein (PV) cells under controls and Pitx2-induced remodelling conditions.*** *At a pacing frequency of 2Hz, AP under control, Pitx2-1, Pitx2-2, Pitx2-3 and Pitx2-4 conditions. Black and red markers were used for LA and PV cells, respectively. Blue arrows indicate spontaneous delayed afterdepolarizations and triggered action potentials.*

*Supplementary Table S2. A quantitative summary of electrophysiology characteristics for left atrial (LA) and pulmonary vein (PV) cells under controls and Pitx2-induced remodelling conditions.*

| | | Control | Pitx2-1 | Pitx2-2 | Pitx2-3 | Pitx2-4 |
|---|---|---|---|---|---|---|
| RMP | LA | -80.97 | -77.45 | -82.05 | -84.38 | -83.23 |
| | PV | -80.69 | -77.18 | -81.39 | -82.71 | -77.80 |
| OS | LA | 24.73 | 22.75 | 24.86 | 25.33 | 27.73 |
| | PV | 24.80 | 22.62 | 24.81 | 25.27 | 24.81 |
| TA | LA | No | No | No | Yes | Yes |
| | PV | No | No | No | Yes | Yes |
| $dV/dt_{max}$ | LA | 209.1 | 193.8 | 211.9 | 215.8 | 231.4 |
| | PV | 207.5 | 190.4 | 209.7 | 213.3 | 206.2 |
| APD | LA | 246.5 | 258.7 | 221.1 | 204.2 | 224.5 |
| | PV | 238.6 | 244.3 | 183.3 | - | 207.3 |
| $\Delta APD$ | LA-PV | 7.9 | 14.4 | 37.8 | - | 17.2 |

*Abbreviations: LA–left atrial cells; PV-pulmonary vein cells; RMP (mV) – resting membrane potential; OS (mV) – overshoot of action potential; dV/dtmax (mV/ms) –maximum upstroke velocity; APD (ms) – action potential duration; and $\Delta APD$ – APD heterogeneity. Under the Pitx2-3 condition, spontaneous delayed afterdepolarizations and triggered action potentials were induced.*

3) The parameter changes for the different Pitx2 cell models should be included in the main body of the text rather than just in the supplement as this is fundamental to the study.

**Response:** We would like to thank this reviewer for his or her detailed suggestions. We have moved the table to the main body of the revised text as the new Table 1. This table lists Pitx2-insufficiency induced remodelling data and model parameters used in our models.

*Table 1. Review of Pitx2-insufficiency induced remodeling data and model parameters in human left atrium.*

| Process | Experimental observation | Control | Pitx2-1 | Pitx2-2 | Pitx2-3 | Pitx2-4 |
|---|---|---|---|---|---|---|
| $I_{Na}$ | -60% (SCN5A & SCN1B) (Chinchilla et al., 2011); -40% (SCN5A & SCN1B) (Lozano-Velasco et al., 2017); +95% SCN5A (Nadadur et al., 2016); No Change (Syeda et al., 2016) | — | — | — | — | +10% |
| $I_{Ks}$ | +150% KCNQ1(Tao et al., 2014); +100% (Pérez-Hernández et al., 2015); Increased volt-dependent potassium current (Kirchhof et al., 2011) | — | — | +100% | — | +150% |
| $I_{K1}$ | -20% (KCNJ2 & KCNJ12) (Chinchilla et al., 2011); +30 (KCNJ2 & KCNJ12) (Lozano-Velasco et al., 2017); -25% (Syeda et al., 2016) | — | -25% | — | — | -30% |
| $I_{CaL}$ | +500% CACNA1D (Tao et al., 2014); -50% (Pérez-Hernández et al., 2015); -50% (Lozano-Velasco et al., 2015); -30% CACNA1C (Lozano-Velasco et al., 2017); Decreased CACNA1C (Kirchhof et al., 2011) | — | — | -50% | -50% | -30% |
| SERCA | +50% ATP2A2(Tao et al., 2014); +1000% ATP2A2(Lozano-Velasco et al., 2015); +100% ATP2A2(Lozano-Velasco et al., 2017); +12% ATP2A2 (Nadadur et al.,2016) | — | — | — | +200% | +100% |
| RyR | +145% RyR2 (Tao et al., 2014); +30% RyR2 (Lozano-Velasco et al., 2015); +30% RyR2 (Lozano-Velasco et al., 2017); +10% RyR2 (Nadadur et al., 2016) | — | — | — | +30% | +30% |
| Gap junctions | -55% GJA1(Chinchilla et al., 2011); -5% GJA1(Nadadur et al., 2016); +100% GJA1 (Tao et al., 2014); +1000% (Pérez-Hernández et al., 2015); -50% (Pérez-Hernández et al., 2015); -58% (Kirchhof et al., 2011) | — | -50% | -50% | -50% | -50% |

*Note: The remodelling in human right atrium is %1 of that in human left atrium. Abbreviations: RyR – ryanodine receptor; SERCA – calcium transport ATPase.*

4) It would be helpful to have more quantitative results in the text and not simply in the figures.

**Response:** We would like to thank this reviewer for his or her detailed comments. In the revised MS, we have added quantitative results: 1) The new Table 2 for summarizing multiple electrophysiology characteristics has been added and 2) Quantitative characteristics have been described in the Result part.

***Table 2. A quantitative summary of electrophysiology characteristics.***

| | | | *Control* | *Pitx2-1* | *Pitx2-2* | *Pitx2-3* | *Pitx2-4* | *Control+F* | *Pitx2-1+F* | *Pitx2-2+F* | *Pitx2-3+F* | *Pitx2-4+F* |
|---|---|---|---|---|---|---|---|---|---|---|---|---|
| *Cell* | *RMP* | *RA* | *-79.89* | *-79.81* | *-79.87* | *-79.97* | *-79.86* | *-81.62* | *-81.59* | *-81.63* | *-81.77* | *-81.67* |
| | | *LA* | *-80.97* | *-77.45* | *-82.05* | *-84.38* | *-83.23* | *-82.10* | *-78.90* | *-83.23* | *-85.94* | *-82.82* |
| | *OS* | *RA* | *24.25* | *24.23* | *24.24* | *24.29* | *24.25* | *24.15* | *24.14* | *24.16* | *24.23* | *24.20* |
| | | *LA* | *24.73* | *22.75* | *24.86* | *25.33* | *27.73* | *24.45* | *22.45* | *24.56* | *24.94* | *27.01* |
| | $dVdt_{max}$ | *RA* | *205.7* | *205.3* | *205.6* | *206.0* | *205.7* | *205.6* | *205.5* | *205.6* | *206.2* | *205.9* |
| | | *LA* | *209.1* | *193.8* | *211.9* | *215.8* | *231.4* | *207.6* | *192.9* | *210.2* | *212.9* | *226.3* |
| | *TA* | *RA* | *No* | *No* | *No* | *No* | *No* | *No* | *No* | *No* | *No* | *No* |
| | | *LA* | *No* | *No* | *No* | *Yes* | *Yes* | *No* | *No* | *No* | *No* | *No* |
| | *APD* | *RA* | *292.5* | *292.7* | *292.0* | *290.6* | *291.5* | *358.9* | *359.1* | *358.3* | *356.8* | *357.5* |
| | | *LA* | *246.5* | *258.7* | *221.1* | *204.2* | *224.5* | *333.4* | *354.9* | *280.8* | *237.5* | *261.6* |
| | *ΔAPD* | | *46.0* | *34.0* | *70.9* | *86.4* | *67.0* | *25.5* | *4.2* | *77.5* | *119.3* | *95.9* |
| *1D* | *CV* | *RA* | *48.61* | *48.62* | *48.60* | *48.59* | *48.63* | *47.24* | *47.27* | *47.25* | *47.23* | *47.28* |
| | | *LA* | *48.48* | *34.56* | *33.27* | *33.01* | *35.59* | *47.22* | *33.46* | *32.45* | *32.25* | *34.53* |
| | *ΔCV* | | *0.13* | *14.06* | *15.33* | *15.58* | *13.04* | *0.02* | *13.81* | *14.8* | *14.98* | *12.75* |
| | *WL* | *RA* | *13.69* | *13.70* | *13.66* | *13.65* | *13.66* | *18.57* | *18.59* | *18.54* | *18.44* | *18.50* |
| | | *LA* | *11.49* | *8.571* | *7.087* | *6.735* | *7.295* | *17.11* | *13.36* | *9.791* | *7.887* | *9.641* |
| | *ΔWL* | | *2.2* | *5.129* | *6.573* | *6.915* | *6.365* | *1.46* | *5.23* | *8.749* | *10.553* | *8.859* |
| | *TA* | | *No* | *No* | *No* | *Yes* | *Yes* | *No* | *No* | *No* | *No* | *No* |
| | $VW_{RA\text{-}LA}$ | | *293.8-294.5* | *324.8-327.8* | *293.8-295.8* | *291.8-298.8* | *291.8-294.8* | *359.8-360.8* | *398.8-402.8* | *332.8-349.8* | *332.8-352.8* | *332.8-349.8* |
| *2D* | *Reentry* | | *-* | *-* | *-* | *-* | *Yes* | *-* | *-* | *-* | *-* | *No* |
| | *TA* | | *-* | *-* | *-* | *-* | *Yes* | *-* | *-* | *-* | *-* | *No* |

*Note: In order to evaluate the RA-to-LA electrical heterogeneity,* $VW_{RA\text{-}LA}$ *of unidirectional conduction block, an index to quantify the RA-to-LA electrical heterogeneity, was quantified by varying the S1-S2 interval in the RA-to-LA strand model with 250 RA myocytes and the other 250 LA cells. The protocol included 10 S1 stimuli applied at the end of the RA part and an S2 stimulus applied at the end segment of the atrial strand.*

**Minor comments:**

Abstract: • "Secondly, our study suggests..." It isn't clear whether this finding is with or without Pitx2 changes, and which of the parameter sets it refers to.

**Response:** Thank you for your careful reading of our manuscript and apologize for the confusion. This finding ("Secondly, our study suggests …") is with Pitx2 deficiency-induced changes (Pitx2-4). The Pitx2-4 condition considers a broad range of experimental data on identified ion channel remodelling (INa, ICaL, IK1, IKs, SERCA, RyR and Gap junctions). In the revised MS, this sentence was modified according to the reviewer's suggestions (Page 2, Lines 18-20):

"*Secondly, our study suggests that flecainide has antiarrhythmic effects on AF due to impaired Pitx2 by preventing spontaneous calcium release and increasing wavelength.*"

Introduction:• "The lack of Pitx2 can alter right-left atrium…" this sentence is very unclear.

**Response:** We would like to thank this reviewer for his or her suggestion and apologize for the confusion. The Pitx2c isoform is the predominant cardiac isoform of Pitx2 and promotes the embryonic development

of cardiac left-right asymmetry, with levels in the right and left atriums being 1:100. Furthermore, PITX2 is essential for the normal development of the sinoatrial node and of the pulmonary vein myocardium. Complete loss of PITX2 function can result in malformation of the pulmonary veins that are well-known sites for ectopic activity, promoting spontaneous AF. In the revised MS (Page 4, Lines 11-14), this sentence was modified according to the reviewer's suggestions:

*"Pitx2 plays an important role in a left-sided signaling pathway that establishes the left-right asymmetry of the heart[7, 8].Pitx2 encodes 3 isoforms (Pitx2a, Pitx2b and Pitx2c) and the Pitx2c isoform promotes the embryonic development of cardiac left–right asymmetry, with levels in right and left atriums being 1:100[9]."*

Results: • Are there are reported differences between Pitx2 level in the appendages or pulmonary veins compared to the atrial body?

**Response**: We would like to thank this reviewer for his or her detailed comments. A previous study showed that the expression levels of PITX2 in the LA are 100-fold higher than those in the RA. However, so far, there are no reports on the differences of the Pitx2 level between the appendages or pulmonary veins and the atrial body.

Results: • Section 3.1 is methods not results. It might be clearer to include the methods section first and then the results section.

**Response:** We would like to thank this reviewer for pointing this out. In the revised MS, we have moved the section 3.1 into the Methods part and put the Methods section (pages 5-8) before the Results section.

Results: • Why did you decide to use 1Hz and 2Hz pacing? Is 2Hz fast enough to test restitution properties? Please could you test the restitution properties of each cell model?

Response: We thank the reviewer for raising this very important question. 1Hz and 2Hz pacing used in our MS is not to test restitution properties, but to examine whether the Pitx2-induced remodelling promotes the generation of triggered activity. Afterdepolarization-triggered arrhythmias are rate dependent, and in general the DAD amplitude increases with decreasing cycle length, causing triggered activity during an increase in heart rate. Therefore, we performed simulations at a normal rate (1Hz) and a fast rate (2Hz).

According to the suggestions of this reviewer, the restitution properties of each cell model are investigated and providd in the new Supplementary Figure S9 in the revised MS.

Action potential duration (APD) restitution curves

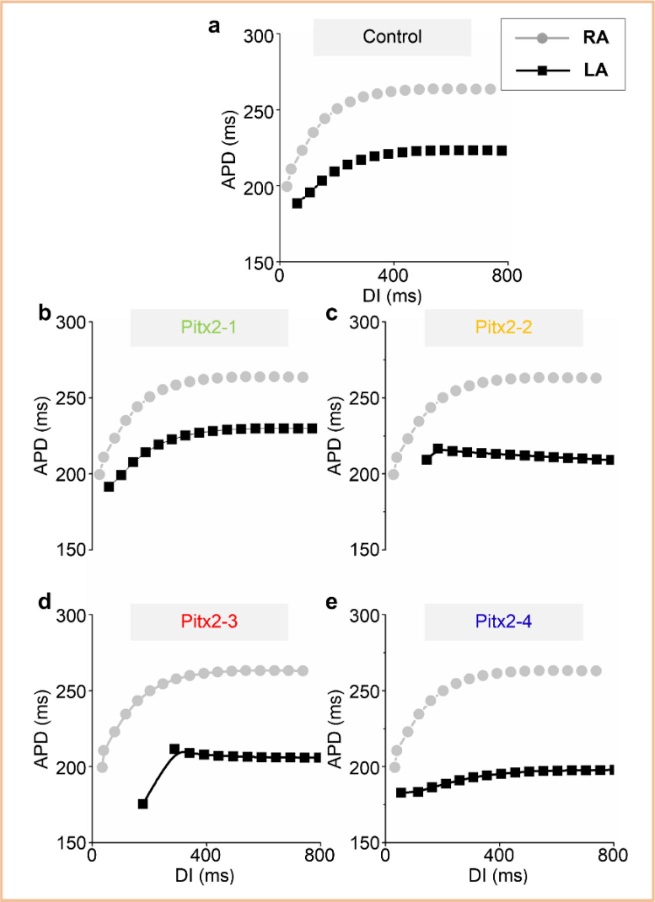


***Supplementary Figure S9. Effects of Pitx2-induced remodelling on action potential duration (APD) restitution properties.*** *(a-e) APD restitution curves for control, Pitx2-1, Pitx2-2, Pitx2-3 and Pitx2-4 conditions. Black and gray markers were used for LA and RA cells, respectively. Abbreviations: APD – action potential duration; DI – diastolic interval; LA – left atrial cell; RA – right atrial cell.*

Results: • The text says heterogeneity in AP features between RA and LA increased to varying degrees, and then later it says differences between RA and LA cells remained unchanged. Please clarify.

**Response:** We apologize for the confusion. Compared to the control condition, heterogeneity in AP features between RA and LA increased to varying degrees under the Pitx2-induced remodelling conditions (drug-free conditions). However, this increased heterogeneity in APD due to Pitx2-induced remodelling was not reduced when these cells (Pitx2-1~Pitx2-4) are treated with flecainide (or the flecainide therapy can not reduce the heterogeneity in AP features between RA and LA caused by Pitx2 deficiency-induced electrical remodelling). In the revised MS, this part has been modified according to improve its clarity (Page 10, Lines 21-24 and Page 11, Lines 1-5):

*"Further simulations were conducted to examine whether the flecainide therapy can reduce the heterogeneity in AP features between RA and LA caused by Pitx2 deficiency-induced electrical remodeling. In presence of 2μM flecainide, our results indicate that heterogeneity in AP features (including AP profile, RMP, overshoot, $dV/dt_{max}$ and $APD_{90}$) between RA and LA increased to varying degrees, compared to those in the absence of flecainide (**Supplementary Figure S4a-e**). With the increase of flecainide concentration within the therapeutic range (0.5-2 μM), $dV/dt_{max}$ decreased and*

*$APD_{90}$ increased in both RA and LA cells. However, their differences between RA and LA cells were not reduced (**Supplementary Figure S4f-g**)."*

Results: • Please include values in the text to support your statements. How much did APD decrease by etc.

**Response:** See our response to your Comment #4.

Results: • How did the Pitx2-3 cells respond to the block effect of flecainide?

**Response**: We would like to thank this reviewer for raising this question. In the revised MS, the new Supplementary Figure S4 shows the antiarrhythmic effects of flecainide on action potentials of LA and RA cells under different conditions (including Pitx2-3). With the increase of flecainide concentration within the therapeutic range (0.5-2 µM), dV/dtmax decreased and APD90 increased in both RA and LA cells.

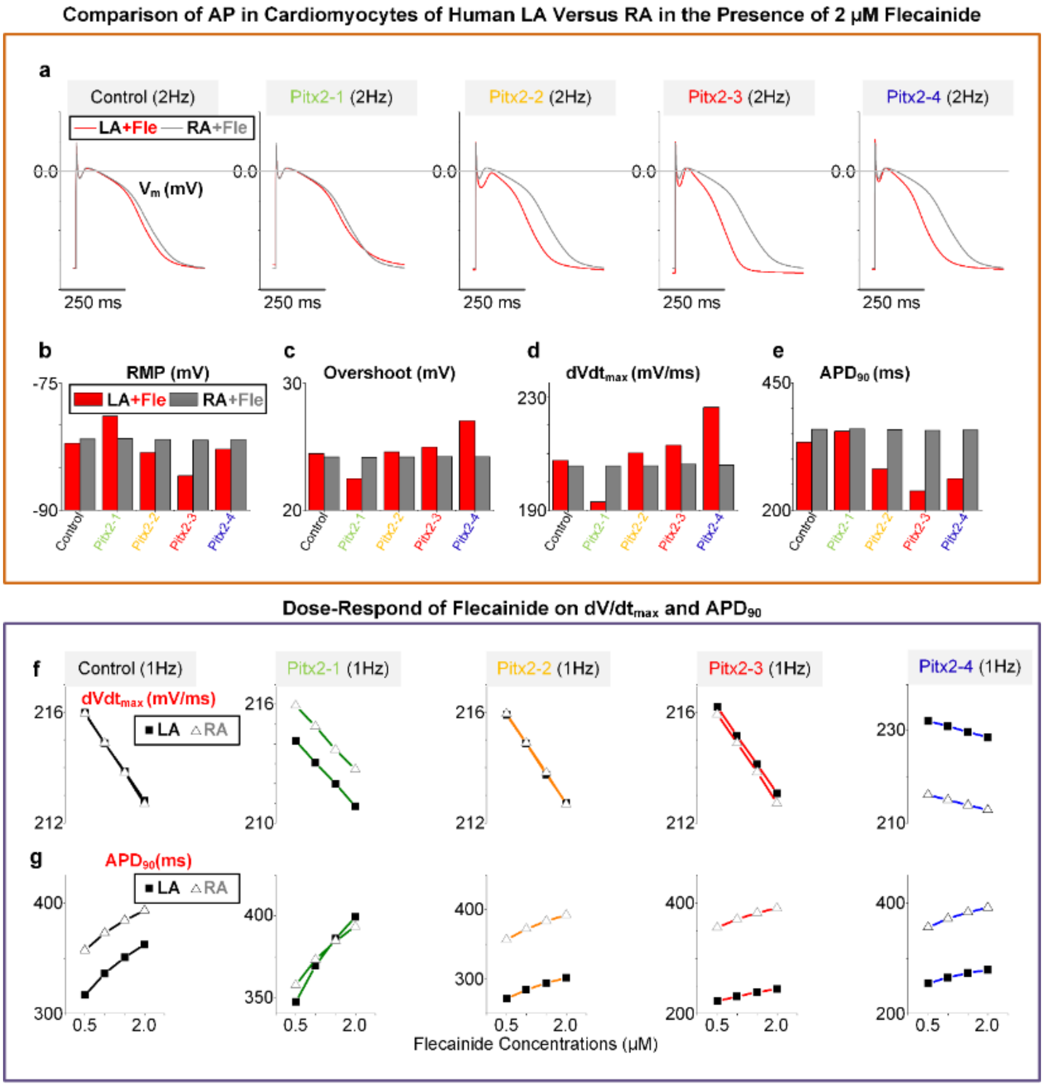


***Supplementary Figure S4. Antiarrhythmic effects of flecainide on action potentials (AP, Vm) of LA and RA cells under control, Pitx2-1, Pitx2-2, Pitx2-3 and Pitx2-4 conditions.** a, Comparison of APs of LA (red) versus RA (gray) cells in the presence of 2µM flecainide under control and four Pitx2-deficiency conditions. The main AP parameters included RMP (b), overshoot (c), dV/dtmax (d) and APD (e). Within clinical dose (0.5~2 µM), flecainide reduced dV/dtmax (f) and prolonged APD (g). Abbreviations: LA – left atrium; RA – right atrium; Fle – flecainide; RMP – resting membrane potential; dV/dtmax –maximum upstroke velocity; APD – action potential duration.*

Results: • It would be very interesting to see how these differences in LA vs RA response affect AF simulations in a realistic bi atrial model.

**Response**: We thank the reviewer for raising this very important question. However, the precise spatial distribution of Pitx2 is unknown, so this important issue is beyond the scope of this study and warrants further study. This limitation has been updated to reflect this in the discussion part (Page 18, Lines 3-13): "*Finally, the precise spatial distribution of Pitx2 throughout human atria was unknown, therefore the regional difference in the cellular properties and remodeling was not investigated. Furthermore, we modelled atrial heterogeneity in the RA-LA tissue by considering the difference in IKr[26]. However, the ionic differences between the RA and LA are more diverse [57, 58] , and this intrinsic heterogeneity should be considered. In addition, our idealized RA-LA tissue model did not include transition regions between RA and LA, electrical properties of transition regions, realistic geometry of these subregions, and fiber orientation. These limitations lead to a sharp transition between the RA and LA in our idealized tissue model. Therefore, special attention should be paid to explain our simulated results, and the effects of these factors on perpetuation and maintenance of re-entrant excitation should be further investigated[50].*"

Discussion:• Key finding 2: Increased fibrosis and decreased cell-to-cell coupling…" Did you show these anchor reentry in this paper?

**Response**: We would like to thank this reviewer for his or her insightful comments. In our simulations, we observed increased fibrosis and decreased cell-to-cell coupling anchored re-entry. In the revised MS, we have added the new *Supplementary Figure 6S* to further explain this finding.

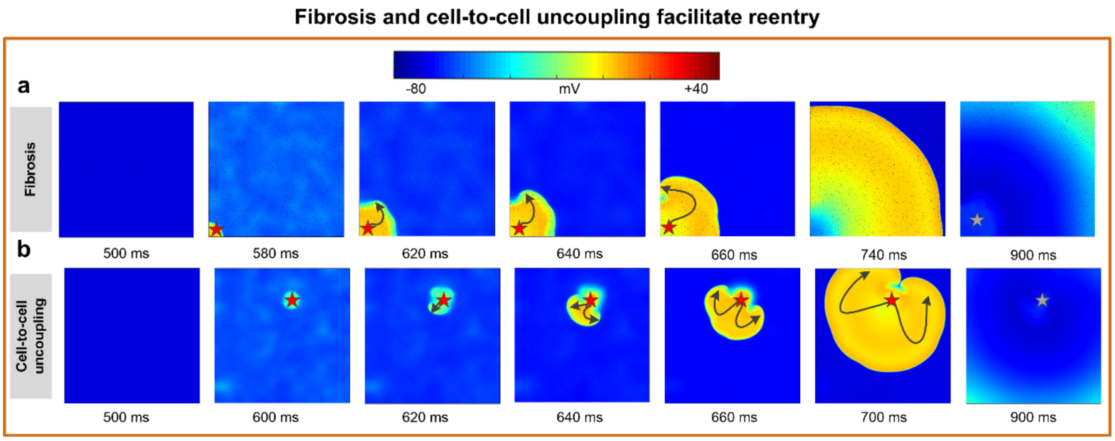


***Supplementary Figure 6S*. *Effects of fibrosis and cell-to-cell uncoupling on ectopic beats. a***, *Simulated spontaneous ectopic activity and re-entrant waves in the tissue model with increased fibrosis.* ***b***, *Simulated ectopic activity and re-entrant waves in the tissue model with cell-to-cell uncoupling*

Discussion • High "accidence" of AF – should be "incidence"

**Response**: Thank you for your careful reading of our manuscript and we have corrected it as suggested.

Discussion • Limitations: Please discuss the choice of cell model.

**Response:** We would like to thank this reviewer for this very constructive suggestion. Although CRN model is based on just as much human measurements, they cannot reproduce DADs due to overloaded $Ca^{2+}$ in

the SR [Fink et al., Am J Physiol Heart Circ Physiol, 2011]. Therefore, the CRN model was modified and validated to generate our basal model (CRN-TPA) developed by our group [Bai et al., Scientific reports, 2018]. The CRN-TPA model was chosen as it is able to reproduce human AP morphology, triggered activity, APD rate dependence and excitation dynamics for studying reentrant arrhythmias in human atrial tissue. In the revised MS, we have discussed the choice of this cell model (Page 17, Lines 5-11):

"*Although the CRN model is based on human measurements, it cannot reproduce DADs due to overloaded calcium in the SR[44]. Therefore, the CRN model was modified and validated to generate our basal model (CRN-TPA) developed by our group[22]. In this study, the modified Grandi model and the CRN-TPA model were chosen as they are able to reproduce human AP morphology, triggered activity (Supplementary Figure S8 and Supplementary Table S2), APD rate dependence and excitation dynamics for studying reentrant arrhythmias in human atrial tissue[22, 45, 46].*"

Discussion • Limitations: The effect of fibrosis modeling methodology has been shown to affect simulated AF dynamics in previous studies.

**Response:** We would like to thank this reviewer for his or her detailed suggestions. In the revised MS, the limitation about fibrosis modelling has been addressed in the discussion part (Page 17, Lines 24-25 and Page 18, Lines 1-3):

*"Thirdly, fibrosis was modeled as nonconducting tissue in this study as consistent with our previous studies[50, 51] and others[52]. In contrast, fibrosis was electrically coupled with healthy myocytes in some modeling studies[53]. Although different methodologies were used to model fibrosis in the past, they all draw the similar conclusions with regard to the role of fibrosis in AF[50, 54-56]."*

Methods:• Please include more details on how this model differs from the original CRN model and also on % changes in conductances for each Pitx2 model.

**Response:** We would like to thank this reviewer for his or her detailed suggestions. In the revised MS, model parameters on % changes in conductances for each Pitx2 model were provided in the Table 1 and Supplementary Table S1. Details on differences between the original CRN model and the basal model (CRN_TPA) developed by our group [Bai et al., Scientific reports, 2018] can be found in our previous published paper and have been added in the revised MS (***Supplementary Figure S1***).

**Supplementary Table S1. Ionic differences in regional cell models.**

| | ICaL(%) | IK1(%) | Ito(%) | IKs(%) | IKr(%) | Source |
|---|---|---|---|---|---|---|
| **RA(Control)** | 100 | 100 | 100 | 100 | 100 | - |
| **LA(Control)** | 100 | 100 | 100 | 100 | 160 | [9, 11] |
| **PV(Control)** | 75 | 70 | 50 | 180 | 100 | [10, 12] |

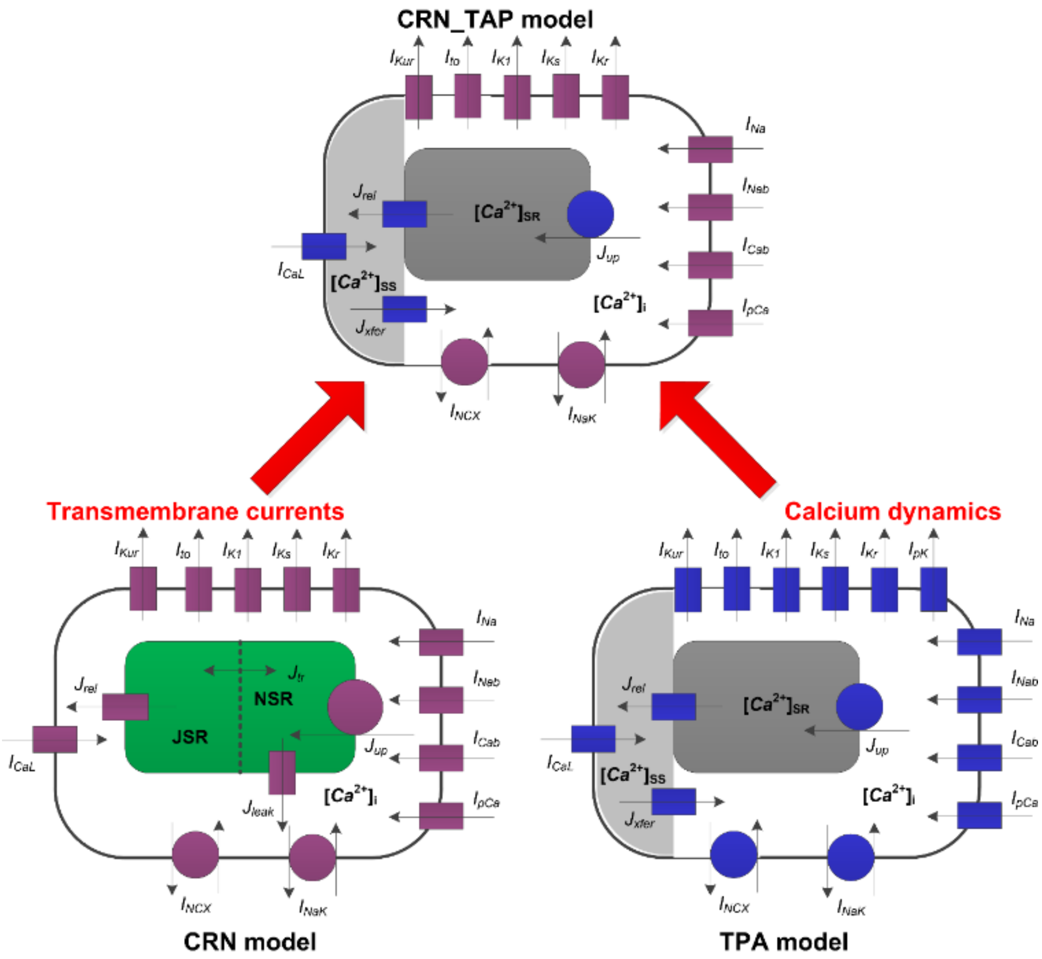


***Supplementary Figure S1. Intracellular structures of the Courtemanche et al. model (CRN), our human atrial (TPA) model and a new human atrial model (CRN_TPA) constructed by integrating the calcium dynamics of our TPA model into the CRN model.*** *The CRN_TPA model was developed by combining the calcium handling formulations from the TPA model and the transmembrane currents of the CRN model. The cell space includes a sub-cellular compartment dyadic cleft (SS), the sarcoplasmic reticulum (SR), the cytoplasm and cell membrane.*

Methods:• Please include a reference for IC50 values.

**Response:** We would like to thank this reviewer for his or her detailed suggestions. In the revised MS, references have been added in the methods part (Page 7, Lines 8-14):

*"Actions of flecainide on ion channels. To investigate the anti-arrhythmic effects of the class Ic drug flecainide, we integrated the actions of flecainide on ion channels and RyR into the Pitx2-mutant computer models. According to experimental/clinical data[24, 28], modifications to ion channels provoked by flecainide were modeled by using the standard sigmoid dose-response curve (Supplementary Figure S2) parametrized with IC50 and Hill coefficient (nH=1). The values of IC50 for inhibition of INa, IKr and RyR open probability were 84, 1.5[28] and 55 µM[24], respectively."*

Methods:• How much did CV change by for the reduced diffusion coefficient simulations?

**Response:** In the revised MS, CV values under different conditions were provided in the Table 2 (See our response to your Comment #3). Compared to the control condition, CV was reduced from 48.61 cm/ms to 34.56 cm/ms when the diffusion coefficient (0.1 cm2/s) was reduced by 50%.

Methods:• What % of cells were normal, Pitx2-4 and fibrosis in the simulations? Did you investigate the sensitivity of simulation outcome to this?

**Response:** We apologize for missing these details. In the revised MS, these details have been added in the Methods part (Page 8, Lines 5-14) and in the Figure 5 legend (Page 23, Lines 7-15). We did not investigate the sensitivity of simulation outcome to these settings.

The Methods part (Page 8, Lines 5-14):

*"To illuminate the spatiotemporal dynamics of triggered activity, a 500×500 square tissue model with randomly distributed normal myocytes, Pitx2-4 cells and fibrosis was designed to simulate synchronization of triggered beats and investigate the conditions of arrhythmia induction. Five different scenarios (#1, #2, #3, #4 and #5) were considered here for investigating effects of Pitx2-4 cells, fibrosis and cell-to-cell uncoupling on the inducibility of ectopic activity. D was set to be 100% for the #1, #2 and #3 conditions, and 30% for the #4 and #5 conditions, respectively. The ratios of normal cells, Pitx2-4 cells and fibrosis were set to be 80:20:0, 40:60:0, 78:20:2, 80:20:0 and 78:20:2, for the #1, #2, #3, #4 and #5 conditions, respectively. AP synchronization in the tissue model was simulated by a stimulus applied to the entire tissue at the beginning according to de Lang's method[31]."*

The Figure 5 legend (Page 23, Lines 7-15):

*"Note: For the #1 scenario, the diffusion coefficient (D) was set to be 100% and the ratio of normal cells, Pitx2-4 cells and fibrosis was set to be 80:20:0. For the #2 scenario, the number of Pitx2-4 cells was increased to 40% and thereby the ratio of different cell types was 40:60:0. And D was set to be 100%. For the #3 scenario, D was reduced to 30% to model cell-to-cell uncoupling and the ratio of different cell types was set to be 80:20:0. For the #4 scenario, fibrosis was increased to 2% and thereby the ratio of different cell types was 78:20:2. And D was set to be 100%. For the #5 scenario, fibrosis was increased to 2% and D was reduced to 30%. And the ratio of different cell types was 78:20:2."*

Methods:• Please include a link to your github.

**Response:** We would like to thank this reviewer for his or her detailed suggestions. Our github have been included in the revised MS (Page 8, Lines 23-24):

"*The electrophysiology codes developed by our team and can be obtained from GitHub (https://github.com/aspirerabbit).*"

Figures:

Figure 1: Make subfigure d larger;

Figure 4: Are any changes significantly different?

Figure 5: Not clear what b-f show?

**Response**: Thank you for your careful reading of our manuscript. We have improved Figure 1 according to your suggestions. The computer models we utilized are not statistical-based approaches, instead they are deterministic models, therefore, these results are significantly different.

For Figure 5, see our response to Reviewer #1 Comment #6.
